# Evaluating aminophylline and progesterone combination treatment to modulate contractility and labor‐related proteins in pregnant human myometrial tissues

**DOI:** 10.1002/prp2.818

**Published:** 2021-07-05

**Authors:** Pei F. Lai, Roger C. Young, Rachel M. Tribe, Mark R. Johnson

**Affiliations:** ^1^ Division of Reproductive and Developmental Biology Department of Metabolism, Digestion and Reproduction Imperial College London London UK; ^2^ PreTeL, Inc. Portland OR USA; ^3^ Department of Women and Children's Health School of Life Course Sciences King's College London London UK

**Keywords:** cAMP, parturition, phosphodiesterase, progesterone, smooth muscle, uterus

## Abstract

Progesterone (P4) and cyclic adenosine monophosphate (cAMP) are regarded as pro‐quiescent factors that suppress uterine contractions during pregnancy. We previously used human primary cells in vitro and mice in vivo to demonstrate that simultaneously enhancing myometrial P4 and cAMP levels may reduce inflammation‐associated preterm labor. Here, we assessed whether aminophylline (Ami; phosphodiesterase inhibitor) and P4 can reduce myometrial contractility and contraction‐associated proteins (CAPs) better together than individually; both agents are clinically used drugs. Myometrial tissues from pregnant non‐laboring women were treated ex vivo with Ami acutely (while spontaneous contracting) or throughout 24‐h tissue culture (±P4); isometric tension measurements, PKA assays, and Western blotting were used to assess tissue contractility, cAMP action, and inflammation. Acute (1 h) treatment with 250 and 750 μM Ami reduced contractions by 50% and 84%, respectively, which was not associated with a directly proportional increase in whole tissue PKA activity. Sustained myometrial relaxation was observed during 24‐h tissue culture with 750 μM Ami, which did not require P4 nor reduce CAPs. COX‐2 protein can be reduced by 300 nM P4 but this did not equate to myometrial relaxation. Ami (250 μM) and P4 (100 and 300 nM) co‐treatment did not prevent oxytocin‐augmented contractions nor reduce CAPs during interleukin‐1β stimulation. Overall, Ami and P4 co‐treatment did not suppress myometrial contractions more than either agent alone, which may be attributed to low specificity and efficacy of Ami; cAMP and P4 action at in utero neighboring reproductive tissues during pregnancy should also be considered.

## Graphical abstract

Aminophylline promoted relaxation and progesterone reduced inflammation, but their combination did not suppress ex vivo human myometrium contractility to mimic uterine quiescence during 24 h treatment. Combination therapy with aminophylline and progesterone, when considering only their direct impact on myometrial contractions, is unlikely to suppress human labour more effectively than either drug alone.

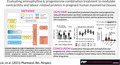

**Abbreviations**
95CI: 95% confidence interval
Ami: aminophylline
BSA: bovine serum albumin
CAP: contraction‐associated protein
COX‐2: cyclooxygenase‐2
CSA: cross‐sectional area
Cx43: connexin‐43
DMEM: Dulbecco's modified Eagle medium
DMSO: dimethyl sulfoxide
DPBS: Dulbecco's phosphate‐buffered saline
GAPDH: glyceraldehyde 3‐phosphate dehydrogenase
H + E: 24 h H_2_O and ethanol (vehicle only) treatment
HSP20: heat shock protein 20
IL‐1β: interleukin‐1β
ITM: isometric tension measurement
KPSS: PSS modified to contain 60 mM KCl
LPS: lipopolysaccharide
MIT: mean integral tension
OTR: oxytocin receptor
P4: progesterone
PDE: phosphodiesterase
PKA: protein kinase A
PR: P4 receptor
PSS: physiological saline solution
PTL: preterm labor
*t* = 0: untreated (no TC) tissues
TC: tissue culture
β2‐AR: β2‐adrenergic receptor

## 1 INTRODUCTION

Optimal fetal development is achieved after 37–40 weeks of human (term) pregnancy. Globally, preterm birth (before 37 weeks of gestation) is a leading cause of death for children under the age of 5^1^ and approximately 70% of all cases occur spontaneously.^2^ Molecular mechanisms responsible for the onset of uterine contractions at labor are not fully established, which has hindered development of effective therapies to prevent preterm labor (PTL).

Labor onset is at least partly related to reduced activity of pathways that suppress myometrial contractile apparatus function and cell‐to‐cell connectivity, which include those regulated by cAMP and progesterone (P4). In myometrium, “P4 functional withdrawal” has been proposed to occur at the end of pregnancy, which involves changes in nuclear P4 receptor (PR) isoform levels and/or function in the absence of a decline in systemic P4 concentrations^3^ to reduce P4‐suppressed transcription of genes for contraction‐associated proteins (CAPs). The ability of cAMP‐driven protein kinase A (PKA) activity to prevent formation of actin‐myosin complexes is expected to decrease at labor to permit phasic contractions; this is supported by previously observed labor‐related changes in expression/activity of cAMP signalling proteins.^4^, ^5^, ^6^

P4 prophylaxis is used for PTL prevention but confidence in its effectiveness is currently limited to pregnant women with premature shortening of the cervix; other risk factors, such as multifetal pregnancy, in the absence of a short cervix are less indicative of acceptable P4 efficacy.^7^, ^8^, ^9^ β2‐adrenergic receptor (β2‐AR) agonists (i.e., betamimetics) that enhance intracellular cAMP concentrations have been used to reduce uterine contractions after PTL has started (i.e., tocolysis) but this has become less common due to maternal side effects.^10^ Drawbacks of betamimetics include myometrial β2‐AR desensitisation,^11^switch in G‐protein coupling^12^and negative feedback by upregulation of cAMP‐phosphodiesterases (PDEs).^13^, ^14^ An alternative route for enhancing myometrial pro‐relaxation effects of cAMP is PDE inhibition.^15^, ^16^, ^17^ Both betamimetics and PDE inhibitors are used as bronchodilators, where they have demonstrated dose‐dependent risks of side effects; however, importantly, their use in combination with steroids has been proposed to improve therapeutic efficacy and reduce side effects in this context.^18^

Previously, we demonstrated that treating pregnant mice with a combination of aminophylline (water‐soluble theophylline; Ami), a PDE inhibitor, and P4 prior to intrauterine injection of lipopolysaccharide (LPS), a pro‐inflammatory inducer of murine PTL, can delay premature pup delivery longer than either agent alone.^19^ Ami alone acutely promotes relaxation of ex vivo human myometrium.^20^ Muscle relaxant effects of Ami are often attributed to its inhibitory action on cAMP‐PDEs that make it a reference drug for bronchodilator design^21^; although it can also act on cGMP‐PDEs, adenosine receptors and histone deacetylases.^22^ Ami is used to treat apnoea^23^ and acute kidney injury^24^ in preterm neonates, which suggests risk of harm related to maternal‐fetal transfer^25^ is low. Maternal tolerance of Ami was found to be better than ritodrine (betamimetic) in a study that also showed their tocolytic efficacies are similar.^26^ Clinical trials for combining betamimetics with other tocolytics to suppress PTL have been conducted^27^ but none published yet for PDE inhibitor and P4 combination therapy.

Here, we examined whether Ami and P4 together suppress human myometrial contractions better than either agent alone, by using a novel method for continuous tension recording during tissue culture (TC), and observed for their effect on PKA to assess extent of cAMP involvement. Our hypotheses were (i) Ami at concentrations that promote myometrial relaxation also increase “total” (i.e., whole tissue) PKA activity, (ii) 24‐h Ami and P4 treatment suppresses spontaneous contractions and enhances pro‐quiescent protein expression/activity more than either agent alone, and (iii) Ami and P4 co‐treatment inhibits interleukin‐1 β (IL‐1β)‐induced changes in PR and CAPs expression/activity more than either agent alone.

## 2 MATERIALS AND METHODS

### 2.1 Materials

Ami from Sigma‐Aldrich for acute treatments and Santa Cruz Biotechnologies for TC experiments. CGS 15943 (adenosine receptor antagonist) from Tocris Bioscience. P4, IL‐1β and dimethyl sulfoxide (DMSO) from Sigma‐Aldrich. Oxytocin from Alliance Pharmaceuticals. Ethanol from Fisher Scientific. Dulbecco's phosphate‐buffered saline (DPBS) and antibiotics (working concentrations of 20 units/ml penicillin and 20 μg/ml streptomycin) from Sigma‐Aldrich. Dulbecco's modified Eagle medium (DMEM; TC media) from ThermoFisher Scientific. Phosphatase inhibitor cocktail 3, β‐mercaptoethanol, Bradford assay reagent and bovine serum albumin (BSA) from Sigma‐Aldrich; cOmplete Mini protease inhibitor cocktail from Roche. SDS‐PAGE and Western blotting consumables from Life Technologies, Bio‐Rad, Millipore and Sigma‐Aldrich; primary and horse radish peroxidase‐conjugated secondary antibodies (Table S1) from Santa Cruz Biotechnologies, Abcam, Millipore and Cell Signalling Technology. Analytical grade reagents for buffer preparations from Fisher Scientific and Sigma‐Aldrich.

### 2.2 Ethical approval and biopsy collection

This study conformed to standards set by the Declaration of Helsinki; ethics approval was granted by the Brompton and Harefield Research Ethics Committee (10/H0801/45), and written consent was provided by all participants prior to sample collection. One myometrium biopsy per woman (*N* = 67) was excised from the upper margin of incision made to the lower uterine segment at term gestation (weeks^+days^ of pregnancy: 39^+0^ median; 37^+1^ to 40^+5^ range) while not in labor during caesarean section for singleton birth (Tables S2 and S3). Biopsies were promptly immersed in DPBS for 2–4°C storage until dissected for isometric tension measurements (ITMs) and/or TC within the same day. Each biopsy was dissected into tissue strips assigned to no more than two different types of experiments (Table S2), the details for which are described in the following subsections and summarized by Figure S1. Dimensions and force measurements of tissue strips are detailed in Tables S4 and S5.

### 2.3 ITM in organ bath without TC

Each biopsy as one biological replicate was dissected into strips of longitudinal muscle in ice‐cold DPBS and tied at both ends with polyester thread to mount onto MLT0201 isometric tension transducers (ADInstruments). After calibration, tissue length was manually adjusted to hold each strip at slack length as defined previously^28^ before immersing in physiological saline solution (PSS; composition described previously^15^) in an 8‐channel organ bath (Panlab). Forces were recorded via transducers connected to a ML870 PowerLab unit and computer operating LabChart 7 (ADInstruments). All buffers were maintained at 37°C with 95% O_2_/5% CO_2_ aeration. Data recording commenced once the last tissue strip of each experiment was lowered into its chamber. After 5 min, 29.4 mN tension was applied to all tissue strips. PSS was changed once every 60 min for the first 3 h while tissues equilibrated and spontaneous contractions stabilized before treatment. Contractions typically initiated within 1.5–2 h after start of data recording, and tissues that failed to produce stable contractions within 3 h were discarded; at least one tissue strip was treated with vehicle and the remaining strips were each treated with drug.

For acute cumulative concentration experiments, the last of these PSS changes was accompanied with the first concentration of Ami (sterile H_2_O vehicle) or CGS 15943 (DMSO vehicle), and each subsequent concentration of both agents was applied at 25‐min intervals without changing PSS. After incubation with the last concentration of Ami or CGS 15943 (0.7% v/v DMSO in total at PSS), PSS at all tissues was exchanged with KPSS (PSS modified to contain 60 mM KCl) to promote maximum tissue depolarisation. After 10 min, KPSS was exchanged with fresh PSS twice and the last of these washes was kept at tissues for at least 10 min, before they were sequentially taken out of their chambers and cut from tied ends to retrieve their sample regions; excess PSS was removed before weighing. Methodology for 1‐h exposure to bolus Ami concentrations was as described previously.^15^

Data analyzed using LabChart 8 (ADInstruments) to calculate mean integral tension (i.e., area under the curve for force vs. time; MIT) and force per contraction; both normalized to cross‐sectional area (CSA)^29^; contraction frequency per 10 min was attained by manual counting of contraction peaks. For cumulative exposure to Ami or CGS 15943, data for each concentration of these agents were obtained from the last 15 min of treatment, and each normalized to the last 15 min of spontaneous contractions immediately prior to first drug application. For 1‐h bolus Ami exposure, data were obtained from the last 30 min of treatment and normalized to the last 30 min of spontaneous activity prior to Ami addition.

### 2.4 TC with simultaneous ITM

Each biopsy as one biological replicate was dissected into four tissue strips to accommodate all 24‐h treatments per experiment; untreated tissues (“*t* = 0”) from each biopsy were dissected at the same time to represent the pre‐culture state and used only for comparison to 24‐h vehicle controls. Dissection in DPBS and attachment to MLT0420 isometric tension transducers (ADInstruments) using polyester thread (pre‐sterilized with ethanol) were undertaken within a biosafety cabinet before transfer into a TC incubator (maintained at 37°C with 95% air/5% CO_2_). Transducers were subsequently connected to a 4SP PowerLab unit and computer operating LabChart 7 (ADInstruments), and calibrated prior to setting all tissue strips to slack length.^28^ Meanwhile, *t* = 0 tissues were briefly stored on ice, while their biopsy‐matched experiment was being set up in the TC incubator, and flash frozen in liquid nitrogen promptly after Ami ± P4 was added to the last tissue strip.

Data recording for each experiment commenced after immersing all tissue strips in DMEM, where 29.4 mN tension was applied after 5 min. Treatments with Ami and P4, along with their vehicles (sterile H_2_O and ethanol (0.02% v/v in DMEM), respectively), were added to tissues immediately after manual stretch stopped. Each Ami, P4 and vehicle combination was mixed in its own sterile DMEM aliquot before adding to its tissue strip using a syringe. Tissue lengths and DMEM were not manually adjusted for the rest of the experiment and the TC incubator was sealed for 24 h of undisturbed data acquisition. After 24 h, transducers were taken out of the TC incubator and tissues were detached to be weighed after removing excess PSS, flash frozen in liquid nitrogen and stored at −80°C.

Data analyzed using LabChart 8 to calculate MIT and basal (i.e., rest period between contractions) tension; both normalized to CSA. These values were taken from the last 1‐h period of every non‐overlapping 4‐h interval of each 24‐h recording (i.e., *T* = 3–4, 7–8, 11–12, 15–16, 19–20, and 23–24 h; *T* = 0 equates to start of data acquisition after setting tissue slack lengths). Data were excluded from biopsies for which none of its tissue strips (irrespective of treatment) sustained regular contractions for at least 1 h within the first 3 h of data recording.

### 2.5 TC for subsequent organ bath ITM (oxytocin stimulation)

Each biopsy as one biological replicate was dissected into six tissue strips in DPBS to accommodate all 24‐h treatments per experiment, and maintained under ~4 mN isotonic tension in TC for 24 h by attaching 0.6–0.7 g mass as described previously.^15^ Ami, P4, and their vehicles (sterile H_2_O and ethanol (0.03% v/v in DMEM), respectively) were added to DMEM immediately before immersing tissues to start 24‐h TC. Samples of *t* = 0 tissues were stored as described for TC with simultaneous ITM experiments.

After 24‐h TC, tissue strips were mounted onto transducers, which were connected to the same organ bath apparatus used for ITM without TC except LabChart 8 was operated for data acquisition; PSS in chambers was supplemented with P4 or its vehicle (ethanol; 0.3% v/v in PSS) immediately before immersing tissues set at slack length. Data recording for each experiment commenced once the last tissue strip was lowered into its chamber. After 5 min, all tissues were stretched to 1.5× slack length and no further manual adjustments were made. Following 2 h with no PSS change, the first concentration of oxytocin (sterile H_2_O vehicle) was added to all tissues and its subsequent concentrations cumulatively applied at 25‐min intervals. Once incubation with the highest oxytocin concentration was complete, all tissue strips were taken out of their chambers to retrieve their sample regions and weighed after removing excess PSS.

Data analyzed using LabChart 8. The same method described for cumulative concentration experiments with Ami and CGS 15943 (without TC) was used, except spontaneous activity was determined from the last 30 min prior to the first application of oxytocin to tissues. Summary score MIT was calculated using a previously published equation.^30^

### 2.6 IL‐1β stimulation during TC without ITM

Each biopsy as one biological replicate was dissected into 12 tissue strips in DPBS to accommodate all 24‐h treatments per experiment. These were maintained under isotonic tension in TC for 24 h in serum‐free DMEM as described for oxytocin stimulation experiments; the same combinations of Ami, P4 and their vehicles were used, except each set of biopsy‐matched tissue strips included treatment with IL‐1β (1 ng/ml) or its vehicle (sterile H_2_O). Samples of *t* = 0 tissues were stored immediately after TC commenced. At 24‐h TC completion, all strips were weighed and flash frozen in liquid nitrogen to store at −80°C.

### 2.7 Total protein extraction & PKA activity assay

Each frozen tissue segment was extracted for total protein content using a Precellys 24 bead‐based homogenizer with CK‐28R tubes (Stretton Scientific) as described previously.^15^ Protein extracts were aliquoted to flash freeze in liquid nitrogen for PKA activity assays or snap frozen on dry ice for protein quantification using Bradford assay reagent (following manufacturer's protocol; BSA standards used) and immunoblotting. PepTag cAMP‐dependent protein kinase assay kit (Promega) was used to measure basal PKA activity as described previously,^15^ when reliability of the assay for myometrial tissues in response to five different cAMP/PKA‐enhancing agents was demonstrated. Agarose gels were imaged using a G:BOX Chemical XL system (Syngene) and analyzed by histogram‐based densitometry using ImageJ v1.5 (National Institutes of Health; Bethesda, MD, USA; Research Resource Identifier (RRID) SCR_003070). Each set of assays included positive (purified PKA catalytic subunit) and background (assay buffer) controls.

### 2.8 Immunoblotting

Total protein extracts (same as those prepared for PKA activity assays) were used as previously described for detecting Ser16‐phosphorylated and total heat shock protein 20 (HSP20),^15^ as well as PR, CAPs (cyclooxygenase‐2 (COX‐2), connexin‐43 (Cx43) and oxytocin receptor (OTR)) and housekeeping proteins (glyceraldehyde 3‐phosphate dehydrogenase (GAPDH) and β‐tubulin)^31^; 10 and 25 μg total protein per sample was used to detect CAPs and PR, respectively. Specificity validation for primary antibodies shown in Figure S2. Western blots were cut to allow simultaneous detection of targets and housekeeping proteins. Chemiluminescence imaging undertaken using a G:BOX Chemical XL system (Syngene). ImageJ v1.5 used for histogram‐based densitometry as described previously.^31^

### 2.9 Data and statistical analysis

Details are provided in Appendix A, figures, and tables.

### 2.10 Nomenclature of targets and ligands

Key protein targets and ligands in this article are hyperlinked to corresponding entries in http://www.guidetopharmacology.org, the common portal for data from the IUPHAR/BPS Guide to PHARMACOLOGY,^32^ and are permanently archived in the Concise Guide to PHARMACOLOGY 2019/20.^33^, ^34^

## 3 RESULTS

### 3.1 Acute Ami exposure on spontaneous contractions and PKA activity

**FIGURE 1:**
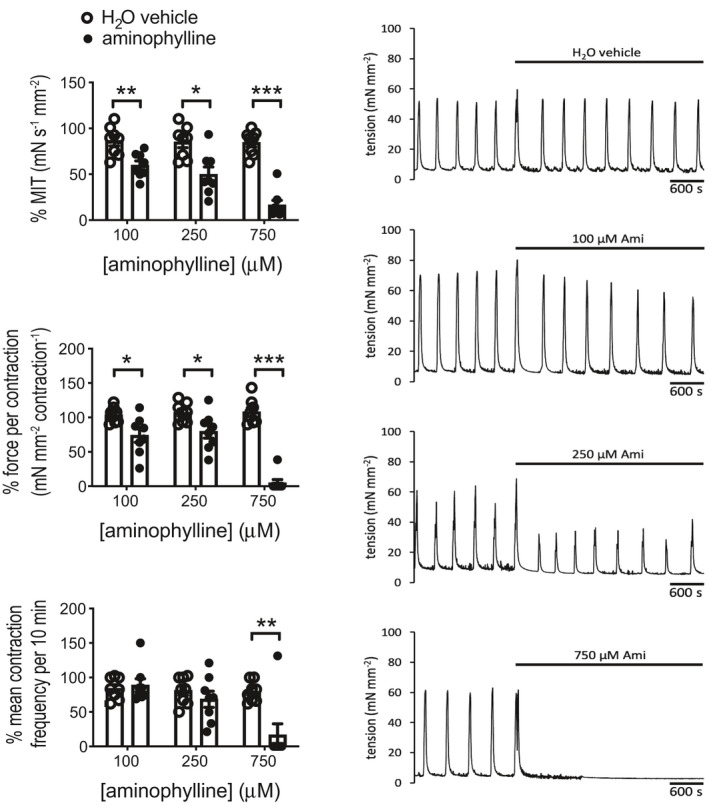
Concentration‐dependent potency of aminophylline for acutely suppressing spontaneous contractions in human myometrial tissues. Isometric tension measurements from myometrial tissues (biopsies from term pregnant non‐laboring women; *N* = 10), which were treated with aminophylline (Ami) or H_2_O vehicle for 1 h after establishing stable spontaneous contractions for ≥1 h. Each biopsy (a biological replicate) was dissected into tissue strips, where one strip was assigned vehicle control and their others treated with one concentration of Ami each; 1‐3 Ami concentrations tested per biopsy, depending on how many produced stable baseline contractions. Data analyzed for mean integral tension (MIT), force per contraction and contraction frequency per 10 min, all for the periods of 30‐min immediately prior to and before the end of Ami treatment for each experiment, which were used to calculate % activity; presented as mean ± SEM, where *n* = 8 for each Ami concentration paired with biopsy‐matched vehicle (left panel). Representative contractility profiles are shown for each Ami concentration, along with vehicle control, all from tissue strips dissected from the same biopsy (right panel). Two‐tailed Mann–Whitney test was used for each pair of biopsy‐matched Ami concentration versus vehicle control comparison; *p ≤ .05, **p ≤ .01, ***p ≤ .001

**FIGURE 2:**
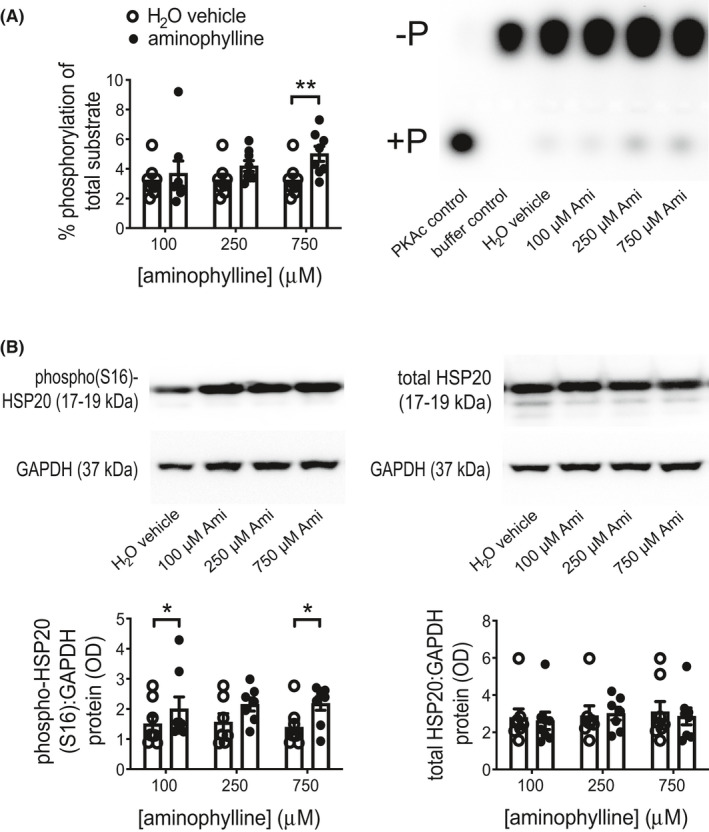
Total PKA activity and HSP20 Ser16‐phosphorylation in human myometrial tissues after 1‐h aminophylline exposure during spontaneous contractions. Total protein extracts were prepared from tissue strips after isometric tension measurements, which were used to assess response to aminophylline (Ami) and its vehicle control during treatment for 1 h (i.e., same tissues represented by Figure 1). These were used for (A) PKA activity assays (purified catalytic PKA subunit (PKAc) used as a positive control and baseline background represented by “buffer control”), and (B) Western blotting for detection of Ser16‐phosphorylated (“phospho(S16)”) and total heat shock protein 20 (HSP20; a PKA substrate) along with glyceraldehyde 3‐phosphate dehydrogenase (GAPDH; loading control). All data presented as mean ± SEM (*n* = 7 for 250 μM Ami, *n* = 8 for 100 and 750 μM Ami; each with biopsy‐matched vehicle controls). Representative images of agarose gels for PKA assays, where “−P” and “+P” indicate non‐phosphorylated and phosphorylated kemptide, respectively, and chemiluminescent Western blots are shown adjacent or above their associated histograms. Two‐tailed paired Student's *t* test (after log transformation for PKA activity data) or Wilcoxon test was used for each biopsy‐matched Ami concentration versus vehicle control comparison; *p ≤ .05, **p ≤ .01

Cumulative Ami treatment demonstrated its inhibitory effect when used after onset of spontaneous contractions; % MITs were used to identify its IC_50_ as 263 μM (95% confidence interval (95CI): 173–388 μM [profile likelihood]; Figure S3). Ami can also inhibit adenosine receptors^35^ but CGS 15943, an adenosine receptor antagonist, at ≤10 μM had no effect on contractions (Figure S4). Bolus Ami treatment for 1 h reduced force generated per contraction relatively more so than contraction frequency (Figure 1). An increase in total PKA activity in these tissues was most observable for 750 μM Ami with a mean fold difference of 1.6 (95CI: 1.2–2.0) compared to vehicle (Figure 2A). HSP20 Ser16‐phosphorylation, which is mediated by PKA and promotes actin depolymerisation,^16^ was increased in 100 and 750 μM Ami‐treated tissues, by mean fold differences of 1.3 (95CI: 1.1–1.6) and 1.7 (95CI: 1.1–2.3), respectively, relative to vehicle (Figure 2B); total HSP20 abundance was unaffected.

### 3.2 Spontaneous contractility during 24‐h Ami ± P4

Ami at 250 and 750 μM gave mean mid‐range (50%; 95CI of 31%–70%) and near‐full (84%; 95CI of 71%–96%) inhibition, respectively, of spontaneous contractions during 1‐h exposure (Figure 1); these concentrations were chosen for assessing Ami effects on spontaneous contractions during 24‐h treatment, with and without P4, using ITM. The rationale was mid‐range inhibition (250 μM) permitted observation of additional or synergistic effects from P4, and near‐full inhibition (750 μM) was useful for observing duration of maximal suppression (according to acute response data) of contractions when applied before their spontaneous onset. P4 at 100 nM maintained a physiologically relevant state at tissue level,^31^ whereas 300 nM was used to model a theoretically therapeutic state that would exist with vaginal progesterone prophylaxis^36^ or alternative (myometrium‐targeted) drug delivery methods.^37^

**FIGURE 3:**
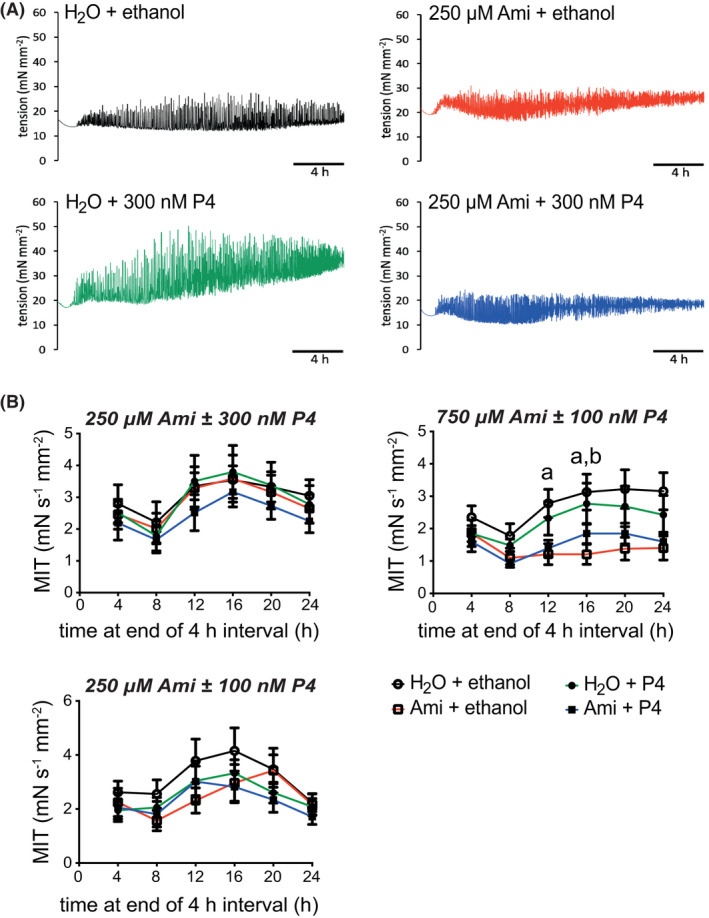
Mean integral tension of spontaneous contractions from human myometrial tissues during 24‐h aminophylline ± progesterone treatment. Isometric tension measurements of spontaneous contractions from myometrial tissues (biopsies from term pregnant non‐laboring women; *N* = 33) during 24‐h treatment with combinations of aminophylline (Ami; H_2_O vehicle) ± progesterone (P4; ethanol vehicle). Each biopsy (as a biological replicate, *n*) was dissected into four tissue strips and assigned to one of three sets of Ami ± P4 treatments, namely, 250 μM Ami ± 300 nM P4 (*n* = 10), 750 μM Ami ± 100 nM P4 (*n* = 11) and 250 μM Ami ± 100 nM P4 (*n* = 12). (A) Representative contractility profiles are shown for each Ami ± P4 combination in a set of 250 μM Ami ± 300 nM P4 treatments for tissue strips from one biopsy. (B) Data for 1 h of mean integral tension (MIT) at the end of every non‐overlapping 4‐h time “interval” during each 24‐h recording, which are presented as mean ± SEM for each Ami ± P4 combination; times on x‐axis are each relative to start of data recording immediately prior to applying 29.4 mN tension to tissues. Kruskal–Wallis test (Dunn's post hoc analysis) was used for comparison of all Ami ± P4 treatments at each time point of interest, and each of the three sets of Ami ± P4 combinations were analyzed separately; ^a^ *p* ≤ .05 for “H_2_O + ethanol” versus “Ami + ethanol”, ^b^ *p* ≤ .05 for “H_2_O + P4” versus “Ami + ethanol”

Together, these Ami and P4 concentrations were used for three sets of experimental conditions for 24‐h treatment with continuous ITM of spontaneous contractions: 250 μM Ami ± 300 nM P4, 750 μM Ami ± 100 nM P4, and 250 μM Ami ± 100 nM P4. From this, Ami was observed to suppress contractions only at 750 μM, which was statistically significant at 12 and 16 h after start of treatment (Figure 3); 750 μM Ami was also associated with a trend towards reduced basal tension (Figure S5). P4 alone or with Ami did not alter contractility during 24‐h ITM in TC, when compared to treatment with only vehicles for Ami and P4 (“H + E” control).

### 3.3 Impact of 24‐h Ami ± P4 on PKA activity, PR, and CAPs

**FIGURE 4:**
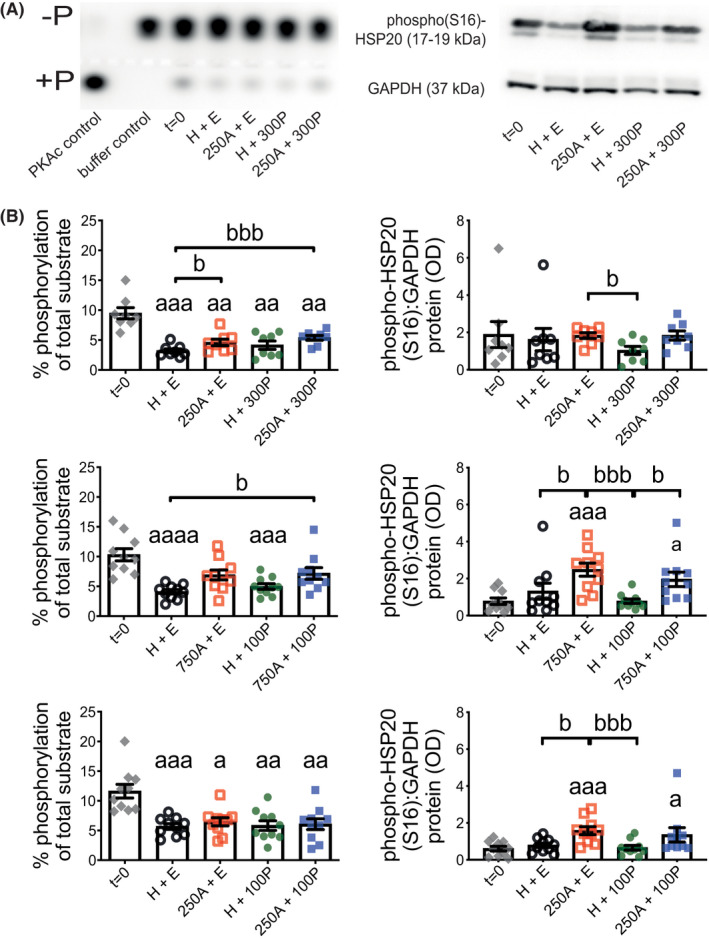
Total PKA activity and HSP20 Ser16‐phosphorylation in human myometrial tissues after 24‐h aminophylline ± progesterone treatment. Total protein extracts were prepared from tissue strips after isometric tension measurements, which were used to assess response at spontaneous contractions to combinations of aminophylline (250 (250A) or 750 (750A) μM; H_2_O vehicle, H) ± progesterone (100 (100P) or 300 (300P) nM; ethanol vehicle, E) during 24‐h treatment in tissue culture media (i.e., same tissues represented by Figure 3); “*t* = 0” (i.e., untreated) biopsy‐matched tissues were also extracted. These were used for PKA activity assays (purified catalytic PKA subunit (PKAc) used as a positive control and baseline background represented by “buffer control”), and Western blotting for detection of Ser16‐phosphorylated (“phospho(S16)”) heat shock protein 20 (HSP20; a PKA substrate) along with glyceraldehyde 3‐phosphate dehydrogenase (GAPDH; loading control). (A) Representative images of agarose gels for PKA assays (left panel), where “−P” and “+P” indicate non‐phosphorylated and phosphorylated kemptide, respectively, and chemiluminescent Western blots (right panel) are shown above their associated histograms for 250A ± 300P treatments. (B) All data presented as mean ± SEM for 250A ± 300P (*n* = 8), 750A ± 100P (*n* = 10) and 250A ± 100P (*n* = 10); *n* equates to number of biopsies for each dataset. PKA activity data were log transformed for repeated measures ANOVA with Geisser–Greenhouse correction (Dunnett's or Tukey's post hoc analysis) for (i) *t* = 0 versus each 24‐h treatment (^a^ *p* ≤ .05, ^aa^ *p* ≤ .01, ^aaa^ *p* ≤ .001, ^aaaa^ *p* ≤ .0001) and (ii) comparisons between 24‐h treatments only (^b^ *p* ≤ .05, ^bbb^ *p* ≤ .001), respectively. Friedman test (Dunn's post hoc analysis) was used for Western blotting data for the same (i) and (ii) comparisons

Total PKA activity measured from whole tissue extracts after 24‐h ITM (Figure 4 and Figure S6) were all decreased relative to biopsy‐matched *t* = 0 (representing untreated (pre‐culture) tissues to closely match the in utero state^38^) except for tissues treated with 750 μM Ami. Less consistency in statistical outcome was observed from comparisons between only 24‐h treated tissues, which showed Ami promoted a trend towards increased total PKA activity when compared to H + E; this effect of Ami was enhanced by P4 for all except 250 μM Ami ± 100 nM P4 conditions. HSP20 Ser16‐phosphorylation in the same tissues (Figure 4 and Figure S6) was increased by Ami at 750 μM to a more discernible extent than 250 μM. While “Ami without P4” versus “P4 without Ami”, irrespective of Ami concentration, consistently showed that a higher amount of Ser16‐phosphorylated HSP20 was present in the former; P4 did not enhance Ami‐associated HSP20 Ser16‐phosphorylation. Total HSP20 abundance (Figure S7) was unchanged by all Ami ± P4 combinations in the same tissues.

**FIGURE 5:**
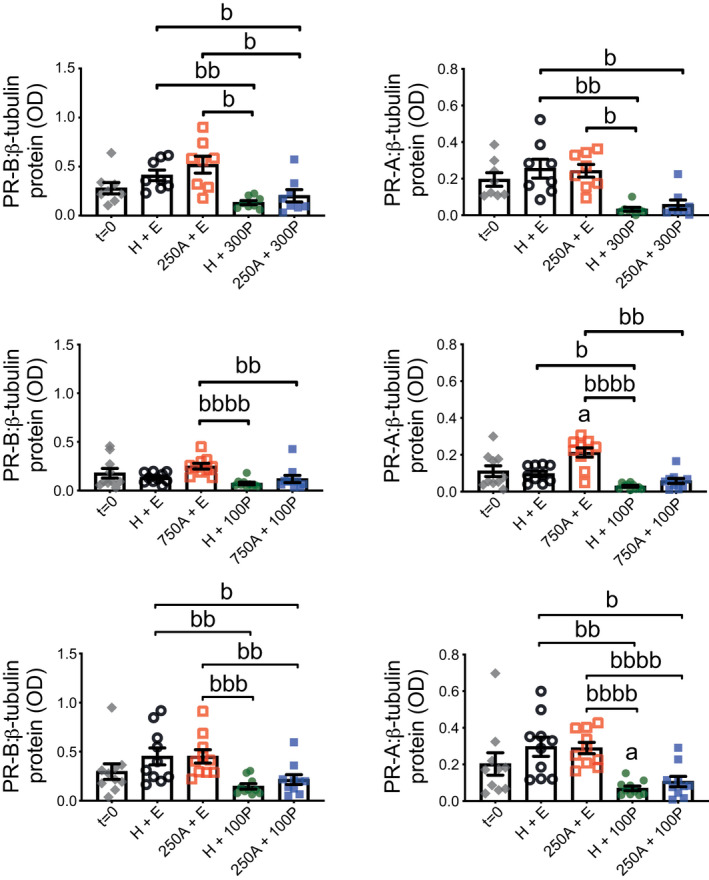
Protein abundance for PR isoforms A and B in human myometrial tissues after 24‐h aminophylline ± progesterone treatment. Total protein extracts were prepared from tissue strips after isometric tension measurements, which were used to assess response at spontaneous contractions to combinations of aminophylline (250 (250A) or 750 (750A) μM; H_2_O vehicle, H) ± progesterone (100 (100P) or 300 (300P) nM; ethanol vehicle, E) during 24‐h treatment in tissue culture media (i.e., same tissues represented by Figure 3); “*t* = 0” (i.e., untreated) biopsy‐matched tissues were also extracted. These were used for detection by Western blotting of progesterone receptor (PR), both isoforms A (PR‐A) and B (PR‐B), and β‐tubulin (loading control); representative images of chemiluminescent Western blots shown at Figure 6 and Figure S8A). All data presented as mean ± SEM for 250A ± 300P (*n* = 8), 750A ± 100P (*n* = 10) and 250A ± 100P (*n* = 10); *n* equates to number of biopsies for each dataset. Repeated measures ANOVA with Geisser‐Greenhouse correction (Dunnett's or Tukey's post hoc analysis) or Friedman test (Dunn's post hoc analysis) was used for (i) *t* = 0 versus each 24‐h treatment (^a^ *p* ≤ .05) and (ii) comparisons between 24‐h treatments only (all pairings; ^b^ *p* ≤ .05, ^bb^ *p* ≤ .01, ^bbb^ *p* ≤ .001, ^bbbb^ *p* ≤ .0001)

**FIGURE 6:**
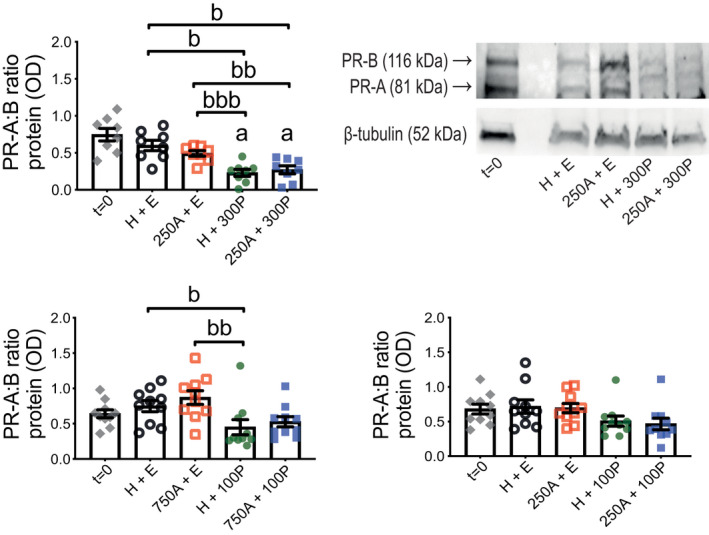
Ratio of PR isoforms A and B at protein level in human myometrial tissues after 24‐h aminophylline ± progesterone treatment. Total protein extracts were prepared from tissue strips after isometric tension measurements, which were used to assess response at spontaneous contractions to combinations of aminophylline (250 (250A) or 750 (750A) μM; H_2_O vehicle, H) ± progesterone (100 (100P) or 300 (300P) nM; ethanol vehicle, E) during 24‐h treatment in tissue culture media (i.e., same tissues represented by Figure 3); “*t* = 0” (i.e., untreated) biopsy‐matched tissues were also extracted. These were used for detection by Western blotting of progesterone receptor (PR), both isoforms A (PR‐A) and B (PR‐B), and β‐tubulin (loading control); representative images of chemiluminescent Western blots are adjacent to its associated histogram for 250A ± 300P and Figure S8A for the other two sets of treatment combinations. Data for individual PR isoforms from which PR ratio values were calculated are shown at Figure 5. All data presented as mean ± SEM for 250A ± 300P (*n* = 8), 750A ± 100P (*n* = 10) and 250A ± 100P (*n* = 10); *n* equates to number of biopsies for whole dataset. Repeated measures ANOVA with Geisser‐Greenhouse correction (Dunnett's or Tukey's post hoc analysis) or Friedman test (Dunn's post hoc analysis) was used for (i) *t* = 0 versus each 24‐h treatment (^a^ *p* ≤ .05) and (ii) comparisons between 24‐h treatments only (all pairings; ^b^ *p* ≤ .05, ^bb^ *p* ≤ .01, ^bbb^ *p* ≤ .001)

PR abundance (Figure 5 and Figure S8A) relative to *t* = 0 was not altered by H + E treatment during 24‐h TC with ITM; PR‐A was increased by 750 μM Ami and, to a lesser extent, decreased by 100 nM P4 relative to *t* = 0. Comparisons between only 24‐h treated tissues showed P4 reduced both PR isoforms regardless of whether Ami was also present. PR ratio (PR‐A:PR‐B; Figure 6 and Figure S8A) was also decreased by P4, which appeared most robust at 300 nM, with and without Ami, when compared to H + E and Ami (without P4); Ami co‐treatment did not increase the ability of P4 to reduce PR‐A:PR‐B.

**FIGURE 7:**
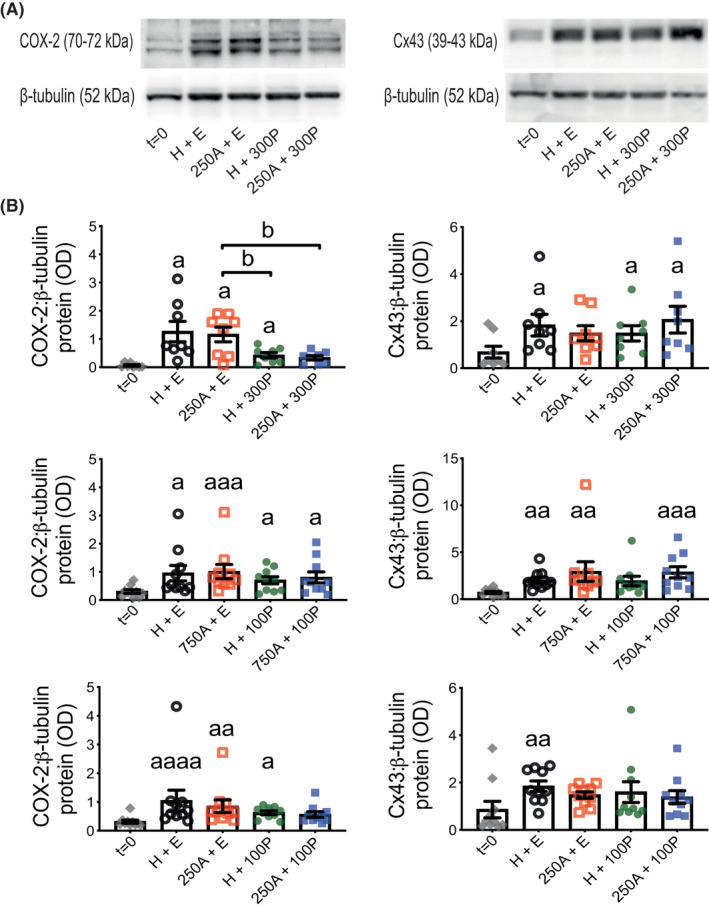
Protein abundance for COX‐2 and Cx43 in human myometrial tissues after 24‐h aminophylline ± progesterone treatment. Total protein extracts were prepared from tissue strips after isometric tension measurements, which were used to assess response at spontaneous contractions to combinations of aminophylline (250 (250A) or 750 (750A) μM; H_2_O vehicle, H) ± progesterone (100 (100P) or 300 (300P) nM; ethanol vehicle, E) during 24‐h treatment in tissue culture media (i.e., same tissues represented by Figure 3); “*t* = 0” (i.e., untreated) biopsy‐matched tissues were also extracted. These were used for detection by Western blotting of cyclooxygenase‐2 (COX‐2) and connexin‐43 (Cx43) along with β‐tubulin (loading control). (A) Representative images of chemiluminescent Western blots for COX‐2 (left panel) and Cx43 (right panel) are above their associated histogram for 250A ± 300P and at Figures S8B and C for the other two sets of treatment combinations. (B) All data presented as mean ± SEM for 250A ± 300P (*n* = 8), 750A ± 100P (*n* = 10) and 250A ± 100P (*n* = 10); *n* equates to number of biopsies for each dataset. Repeated measures ANOVA with Geisser‐Greenhouse correction (Dunnett's or Tukey's post hoc analysis) or Friedman test (Dunn's post hoc analysis) was used for (i) *t* = 0 versus each 24‐h treatment (^a^ *p* ≤ .05, ^aa^ *p* ≤ .01, ^aaa^ *p* ≤ .001, ^aaaa^ *p* ≤ .0001) and (ii) comparisons between 24‐h treatments only (all pairings; ^b^ *p* ≤ .05)

COX‐2 and Cx43 abundance after 24‐h TC with ITM were measured to represent myometrium P4‐sensitive CAPs^39^, ^40^ (Figure 7 and Figure S8B and C). COX‐2 was increased in all 24‐h treated tissues relative to *t* = 0 except for those incubated with 250 μM Ami and either 100 or, arguably more so, 300 nM P4. Comparison between only 24‐h treated tissues showed 300 nM P4, with and without Ami, robustly reduced COX‐2 levels when compared to 250 μM Ami (without P4); the latter was not notably different to H + E. Cx43 was mostly increased during 24‐h ITM in TC when compared to *t* = 0, but there were no differences between only Ami ± P4 combinations (Figure 7 and Figure S8C). OTR was another CAP measured for its abundance in the same tissues (Figure S9) but showed no differences from the same comparisons tested for COX‐2 and Cx43.

### 3.4 Oxytocin response after 24‐h Ami ± P4

**FIGURE 8:**
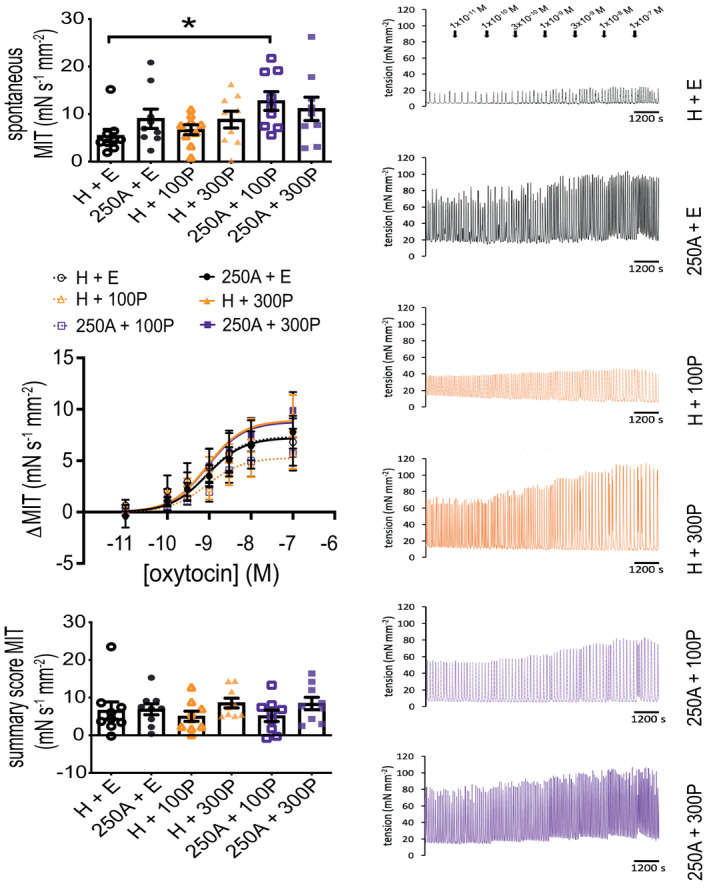
Oxytocin‐stimulated contractile output of human myometrial tissues after 24‐h aminophylline ± progesterone treatment. Isometric tension measurements to measure contractile response to oxytocin stimulation of myometrial tissues (biopsies from term pregnant non‐laboring women; *N* = 9) after 24‐h treatment with combinations of aminophylline (250 μM (250A); H_2_O vehicle, H) ± progesterone (100 (100P) or 300 (300P) nM; ethanol vehicle, E) in serum‐free culture media while maintained under isotonic tension (~4 mN). After 24‐h A ± P treatment, tissue strips were measured for spontaneous activity for 2 h prior to stimulation with oxytocin applied in a cumulative manner (once every 25 min). Data analyzed for mean integral tension (MIT) of spontaneous contractions in the last 30‐min period immediately prior to applying first (i.e., 1 × 10^−11^ M) oxytocin treatment, and change in contractile output after cumulative stimulation with oxytocin relative to spontaneous MIT (ΔMIT; each oxytocin concentration represented by the last 15‐min of its treatment period); summary score MIT was calculated from the latter. All data presented as mean ± SEM (*n* = 9; left panel); *n* equates to number of biopsies for each dataset. Representative contractility profiles (right panel) are shown for each A ± P combination applied to tissue strips from one biopsy. Kruskal–Wallis test (Dunn's post hoc analysis) was applied to spontaneous MIT and summary score (oxytocin stimulation) MIT data for comparison of all possible treatment pairings; *p ≤ .05. The log[agonist] versus normalized response (three parameters) model was used for curve fitting by non‐linear regression of ΔMIT data; extra sum‐of‐squares *F* test was used to compare logEC_50_ values between all 24‐h A ± P treatments, *p* = .79

Tissues treated with both 250 μM Ami and 100 nM P4 during 24‐h TC with isotonic tension were subsequently the most spontaneously contractile (Figure 8). Oxytocin was used to assess whether 24‐h Ami ± P4 can alter myometrial response to hormone‐augmented contractions; no differences in contractility during cumulative exposure to oxytocin (normalized to pre‐oxytocin spontaneous activity) were observed between all Ami ± P4 combinations used for 24‐h TC; oxytocin EC_50_ values are provided at Table 1.

**TABLE 1:** EC_50_ for oxytocin‐augmented myometrial contractility (ΔMIT) after 24 h tissue culture with Ami ± P4

24 h Ami ± P4 treatment during tissue culture with ~4 mN isotonic tension	Oxytocin EC_50_ (M)	Lower 95% CI for oxytocin EC_50_ (M)	Upper 95% CI for oxytocin EC_50_ (M)	Oxytocin log EC_50_
H_2_O + EtOH	3.69 × 10^−10^	2.38 × 10^−11^	2.47 × 10^−9^	−9.43
250 μM Ami + EtOH	1.04 × 10^−9^	3.04 × 10^−10^	3.09 × 10^−9^	−8.98
H_2_O + 100 nM P4	1.00 × 10^−9^	1.62 × 10^−10^	4.14 × 10^−9^	−9.00
H_2_O + 300 nM P4	8.73 × 10^−10^	3.52 × 10^−10^	1.99 × 10^−9^	−9.06
250 μM Ami + 100 nM P4	1.49 × 10^−9^	3.69 × 10^−10^	4.81 × 10^−9^	−8.83
250 μM Ami + 300 nM P4	1.14 × 10^−9^	2.94 × 10^−10^	3.76 × 10^−9^	−8.95

Abbreviations: Ami, aminophylline; CI, profile likelihood confidence intervals; EtOH, ethanol (P4 vehicle); MIT, mean integral tension; P4, progesterone.

### 3.5 IL‐1β influence on Ami ± P4

**FIGURE 9:**
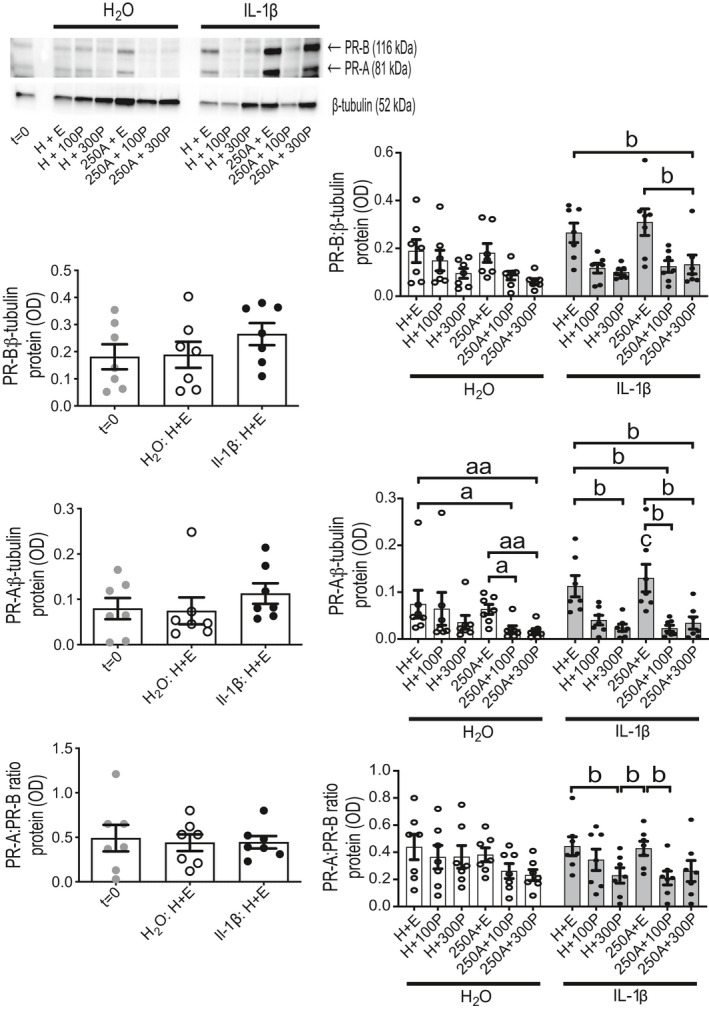
Protein abundance and ratio for PR isoforms A and B in human myometrial tissues after 24‐h aminophylline ± progesterone ± IL‐1β treatment. Total protein extracts were prepared from tissue strips after treatment in serum‐free culture media with combinations of aminophylline (250 μM (250A); H_2_O vehicle, H) ± progesterone (100 (100P) or 300 (300P) nM; ethanol vehicle, E) ± interleukin‐1β (1 ng/mL in H_2_O vehicle; IL‐1β) for 24 h while maintained under isotonic tension (~4 mN); “*t* = 0” (i.e., untreated) biopsy‐matched tissues were also extracted. These were used for detection by Western blotting of progesterone receptor (PR), both isoforms A (PR‐A) and B (PR‐B), and β‐tubulin (loading control); representative images of chemiluminescent Western blots are shown for tissue strips from one biopsy. All data presented as mean ± SEM (*n* = 7); *n* equates to number of biopsies for each dataset. Repeated measures ANOVA with Geisser‐Greenhouse correction (Dunnett's or Tukey's post hoc analysis) or Friedman test (Dunn's post hoc analysis) was used for (i) *t* = 0 versus 24‐h treatment with IL‐1β or its vehicle control (both also with vehicles for A and P, all *p* > .05; left panel), and (ii) comparisons between only 24‐h treatments with either IL‐1β (^b^ *p* ≤ .05) or its vehicle control (^a^ *p* ≤ .05, ^aa^ *p* ≤ .01) for all possible pairings (right panel); two‐way repeated measures ANOVA (Sidak's post hoc analysis) was used for IL‐1β versus its vehicle control comparison for matched A ± P treatments (^c^ *p* ≤ .05)

Tissues treated with IL‐1β or its vehicle, with the same Ami ± P4 combinations used to test oxytocin response (Figure 8), during 24‐h TC with isotonic tension were analyzed for change in response to this pro‐inflammatory cytokine. PR‐A and PR‐B abundance, along with their ratio, were not different between H + E, with and without IL‐1β, and *t* = 0 (Figure 9); whereas comparison between only 24‐h treatment conditions showed P4 (100 and 300 nM), especially with Ami but irrespective of IL‐1β co‐treatment, reduced PR‐A abundance, when compared to H + E and Ami (without P4). A similar observation was made for PR‐B but this was only notable for 250 μM Ami and 300 nM P4 co‐treatment with IL‐1β. Two‐way ANOVA for IL‐1β versus its vehicle showed a difference for Ami without P4, where IL‐1β increased PR‐A. Together, these changes to PR corresponded to decreased PR‐A:PR‐B associated with 300 nM P4 (without Ami), only in the presence of IL‐1β, when compared to H + E or Ami (without P4); comparison of Ami and 100 nM P4 co‐treatment to the latter showed PR‐A:PR‐B was reduced.

**FIGURE 10:**
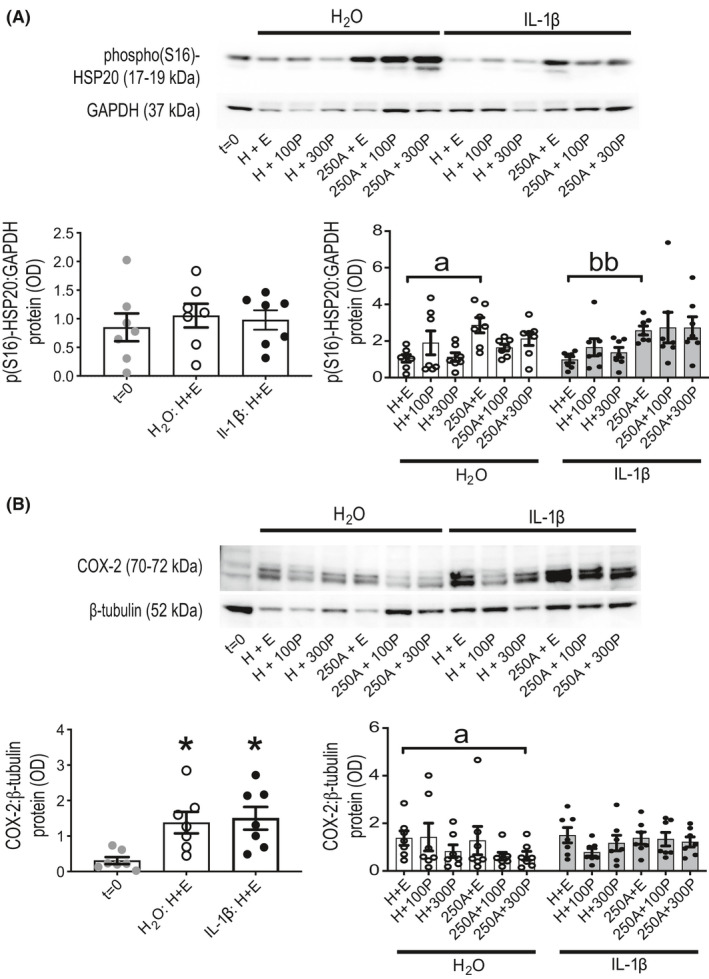
Protein abundance for HSP20 (Ser16‐phosphorylated) and COX‐2 in human myometrial tissues after 24‐h aminophylline ± progesterone ± IL‐1β treatment. Total protein extracts were prepared from tissue strips after treatment in serum‐free culture media with combinations of aminophylline (250 μM (250A); H_2_O vehicle, H) ± progesterone (100 (100P) or 300 (300P) nM; ethanol vehicle, E) ± interleukin‐1β (1 ng/ml in H_2_O vehicle; IL‐1β) for 24 h while maintained under isotonic tension (~4 mN); “*t* = 0” (i.e., untreated) biopsy‐matched tissues were also extracted. These were used for detection by Western blotting of (A) Ser16‐phosphorylated (“phospho(S16)” and “p(S16)”) heat shock protein 20 (HSP20, a PKA substrate; top panel) and (B) cyclooxygenase‐2 (COX‐2; bottom panel), along with glyceraldehyde 3‐phosphate dehydrogenase (GAPDH) and β‐tubulin (loading controls). Representative images of chemiluminescent Western blots are shown for biopsy‐matched tissue strips above their associated histograms. All data presented as mean ± SEM (*n* = 7); *n* equates to number of biopsies for each dataset. Repeated measures ANOVA with Geisser‐Greenhouse correction (Dunnett's post hoc analysis) was used for *t* = 0 versus 24‐h treatment with IL‐1β or its vehicle control (both also with vehicles for A and P, *p ≤ .05; left panel). Friedman test (Dunn's post hoc analysis) was used for comparisons between only 24‐h treatments with either IL‐1β (^bb^ *p* ≤ .01) or its vehicle control (^a^ *p* ≤ .05) for all possible pairings (right panel); two‐way repeated measures ANOVA (Sidak's post hoc analysis) was used for IL‐1β versus its vehicle control comparison for matched A±P treatments (all *p* > .05)

Total PKA activity decreased during 24‐h TC in H + E controls, irrespective of whether IL‐1β was also present, when compared to *t* = 0; whereas it was unaffected by all Ami ± P4 ± IL‐1β combinations when only 24‐h treatments were compared to each other (Figure S10). HSP20 Ser16‐phosphorylation levels were not different between *t* = 0 and 24‐h H + E controls, irrespective of IL‐1β co‐treatment; whereas comparison between only 24‐h treated tissues showed that it was increased by Ami (without P4) relative to H + E irrespective of IL‐1β condition (Figure 10A); total HSP20 abundance was not different for all comparisons (Figure S10). COX‐2 abundance was increased in H + E controls, both with and without IL‐1β, when compared to *t* = 0 (Figure 10B). COX‐2 was also decreased in the absence of IL‐1β, but not in its presence, by 250 μM Ami and 300 nM P4 co‐treatment when compared to H+E. Cx43 and OTR levels were also measured but no differences were found from the same comparisons described for Ser16‐phosphorylated HSP20 and COX‐2 (Figure S11).

## 4 DISCUSSION

This study validated previous observations of Ami's acute pro‐relaxation effects on ex vivo human myometrium.^20^ Additionally, our data suggest that acute Ami‐mediated relaxation does not directly correlate with PKA activity at whole tissue level. This is theoretically consistent with findings from cardiovascular/respiratory muscle research, which often show that cAMP‐PDE isoforms exert their multitude of actions within discrete intracellular microdomains^41^; such compartmentalisation was destroyed in our tissue extracts so that we only measured total PKA activity. We previously used the same experimental approaches to observe acute effects of five cAMP/PKA‐enhancing agents,^15^ one of which was rolipram (archetypal cAMP‐PDE inhibitor; specific to the PDE4 family) and its data were most similar to that from Ami (inhibits PDE3 and PDE4 families^22^). In the present study, we also identified an Ami concentration (750 μM) that inhibits contractions in an acute tocolytic manner for 1 h and promotes relaxation during 24‐h TC. However, Ami's effects were not improved by P4, and their co‐treatment in the presence of IL‐1β did not increase total PKA activity nor decrease CAPs abundance more than either agent alone.

Previously, P4 has been shown to increase cAMP content in myometrial cells and tissue extracts.^42^, ^43^, ^44^ P4 in vivo can maintain myometrial PKA activity at the plasma membrane, where it is most effective in suppressing contractile apparatus function.^45^, ^46^ Corticotrophin‐releasing hormone can upregulate myometrial cAMP synthesis^47^ but its pro‐relaxation effects are only notable when P4 is present.^48^, ^49^ Myometrial inflammation (associated with labor) can be regulated by cAMP and P4.^50^ We previously showed that an adenylate cyclase activator, forskolin, can increase PR‐B expression and activity in primary human myometrial cells.^51^ Amini et al. has since demonstrated PR‐A Ser345‐phosphorylation, which is increased by IL‐1β to enhance its trans‐repressive effect on PR‐B, can be reduced by forskolin in a PR‐expressing human myometrial smooth muscle cell line.^52^ Taken together, these observations suggest that enhancing myometrial P4 and cAMP signalling together prevents labor onset; whether this could be achieved using a clinically approved PDE‐inhibiting drug (capable of enhancing cAMP) with P4 on human tissues had yet to be determined.

Here, we used novel 24‐h ITM methodology to monitor ex vivo human myometrial contractions for real‐time assessment of physiological changes in response to Ami ± P4. From this, we observed no discernible impact except for Ami at a concentration that is not clinically feasible (750 μM) but nevertheless aided proof of concept. Ami at 250 μM neither prevented contractions nor robustly promoted total PKA activity and HSP20 Ser16‐phosphorylation; previous bioavailability data^53^, ^54^ suggests this Ami concentration is also not clinically feasible unless myometrium‐targeted drug delivery methods are used.^37^ Ami is a weak pan‐PDE inhibitor with a relatively poor therapeutic index^55^ and we have now demonstrated its weakness as a long‐lasting myometrial relaxant. Isoform‐selective cAMP‐PDE inhibitors with better pharmacokinetic profiles^56^ should be assessed to determine whether Ami's weakness for sustaining myometrial relaxation, with or without P4, are unique to Ami or truly applicable to all cAMP‐PDE inhibitors; 43 cAMP‐PDE isoforms exist^56^ although only some will be expressed in human myometrium.^57^

Although we had previously observed Ami combined with P4 suppresses LPS‐induced preterm pup delivery in mice,^19^ we had not monitored in vivo intrauterine pressure. We also had not analyzed the placenta and cervix of Ami ± P4 treated mice. In contrast, Schmitz et al. had previously shown that rolipram can reduce LPS‐driven preterm pup delivery, which was associated with changes at the decidual‐placental interface, amniotic fluid and cervix.^58^ Thus, another possible reason why Ami with P4 had little impact on human myometrium was because their combined effects required the presence of other reproductive tissues, and their absence in our experimental model limits our ability to interpret this further.

P4 has been proposed to suppress myometrial contractions by reducing COX‐2.^59^, ^60^ We observed P4 treatment (± Ami) during 24‐h ITM in TC did not suppress contractions. Simultaneously, P4 was able to reduce COX‐2 without affecting Cx43 and OTR, which are (in principle) better CAPs for correlation with myometrial function because they have less ambiguous contraction‐related roles.^61^ Overall, P4‐driven reduction of COX‐2 protein alone does not suppress contractions. Thus, changes in abundance of “labor‐related” proteins does not necessary equate to expected changes in physiological output of myometrium, the contractility of which is expected to be driven by a plethora of protein interactions.

P4 functional withdrawal is associated with PR‐A dominance and increased inflammation.^62^ However, we did not observe increased PR‐A:PR‐B in 24‐h TC vehicle controls despite enhanced COX‐2 abundance relative to *t* = 0 and even in the presence of IL‐1β.^63^ Furthermore, P4 (300 nM) reduced PR‐A:PR‐B in IL‐1β‐treated tissues without affecting COX‐2 abundance. Together, these findings suggest that PR‐A dominance is not a necessity for COX‐2 up‐regulation and IL‐1β may stabilize COX‐2 more so than PR‐A in ex vivo myometrium.

In agreement with our previous study,^31^ 24‐h TC with vehicle controls can increase COX‐2 and Cx43 abundance, when compared to *t* = 0. PR was previously found to decrease after 24‐h TC with only vehicle treatment^31^ but unaffected in the present study; the vehicles used were different between these studies, which may responsible for this inconsistency. Within the present study, isometric and isotonic tension experiments did not always produce the same results, especially for comparisons between only 24‐h treatments. Future use of ex vivo mechanical stretch to mimic pregnancy should explicitly consider whether type/intensity of biomechanical stress can influence outcome of drug screening studies.

One of our study limitations was the use of biopsies from term pregnancies. Myometrium biopsies from preterm pregnancies may have produced findings deemed more relevant to evaluating Ami and P4 efficacy for PTL prevention. Preterm and term non‐laboring women comparisons have previously indicated that their myometrial transcriptomes are not notably different,^64^, ^65^ although may be distinct at gene ontology analysis^66^; how this translates to differences in physiological output is undetermined. We were restricted to using biopsies from term caesarean sections because they were more frequent than preterm, which made achieving necessary biological replicates for each experiment more attainable. Although this limits data interpretation in the context of PTL, the experiments were still valuable for what was a novel evaluation of Ami and P4 co‐treatment on myometrial contractions (as a functional output of all labor), and they were needed for us to begin optimising methodology for 24‐h tension recordings before use with less readily available preterm biopsies.

Another study limitation was the absence of experiments for quantifying cAMP concentrations. Enzyme immunoassay‐based cAMP quantification may have provided more certainty to Ami's effectiveness as a cAMP‐PDE inhibitor, by distinguishing this from its effects on cGMP‐PDEs (especially given that cGMP‐activated protein kinase G can also increase HSP20 Ser16‐phosphorylation^67^) and activities unrelated to cyclic nucleotides.^22^ However, this would still only determine total cAMP action; relevance of microdomain cAMP signalling for Ami‐driven relaxation requires methods that interrogate subcellular compartments,^68^ which were not available for the present study but should, where possible, be used for future myometrium‐based evaluation of cAMP‐PDEs as drug targets. Additionally, Ami and P4 quantification after TC would have helped to determine whether their low efficacy was attributed to poor drug stability; nevertheless, ITM experiments showed Ami's weakness as a pro‐relaxant, which was notable within 1 h of acute treatment, and Ami and P4 effects on contractions did not demonstrate a time‐dependent decline to indicate progressive reduction in their stability during 24‐h TC.

In conclusion, our novel 24‐h ITM recording system for ex vivo myometrial tissues has helped to demonstrate that Ami and P4 together do not prevent contractions in human myometrium better than either agent alone. However, we cannot exclude the possibilities that (i) alternative cAMP‐PDE inhibitors could suppress myometrial contractions more effectively, (ii) Ami and P4 combination therapy can inhibit events in other reproductive tissues that influence labor status, and (iii) a different outcome would be observed in preterm myometrial tissues. These factors will need to be addressed in future investigations to improve evaluation of cAMP and P4 combination therapy for PTL prevention.

## DISCLOSURE

The authors have none to declare for this study.

## ETHICS APPROVAL STATEMENT

Ethics for this study was approved by the Brompton and Harefield Research Ethics Committee (London, UK); reference number 10/H0801/45.

## AUTHOR CONTRIBUTIONS

Study conceptualisation by PFL and MRJ; study design by PFL and RCY; all experiments, data analysis and writing of original draft manuscript undertaken by PFL; data interpretation, along with review, editing and approval of the manuscript’s final version, by PFL, RCY, RMT and MRJ.

## Supporting information

Fig S1‐S10

Table S1‐S5

## Data Availability

The data that support the findings of this study are available from the corresponding author upon reasonable request. Some data may not be made available because of privacy or ethical restrictions.

## APPENDIX A

### DATA AND STATISTICAL ANALYSIS

Assessment of fit to normal distribution and statistical significance of comparisons between groups (each consisting of values only from biological replicates), along with curve fitting by non‐linear regression of contractility data from cumulative exposure treatments, were undertaken using Prism v8.4 (GraphPad; RRID SCR_002798). Statistical comparisons implemented were planned based on predefined hypotheses before data collection. Differences were deemed statistically significant where *p* ≤ .05, and *p* values from comparisons between ≥3 groups were adjusted for multiplicity of pairings. Low concentrations of total protein extracts for some tissues meant that all conditions for their biopsy were not used for PKA activity assays and Western blotting, hence why their *n* numbers at data analysis were lower than those of their associated ITM experiments.

Shapiro‐Wilk (where *n* = 6–7) or D'Agostino Pearson (where *n* ≥ 8) normality tests were used to determine suitability of non‐/parametric statistical analysis for patient demographics, tissue dimension parameters and each protein target of interest analyzed from Western blots; specific statistical tests and, where appropriate, ANOVA‐associated post hoc analyses for these types of data are stated in Table footnotes and Figure legends. Mann–Whitney test and Kruskal–Wallis with Dunn's post hoc analysis were used for all contractility data. Student's paired *t* test or repeated measures ANOVA with Geisser‐Greenhouse correction and either Dunnett's or Tukey's post hoc analysis was used for PKA activity data, which was log transformed beforehand when not normally distributed.

Log[inhibitor] versus normalized response (variable slope) and log[agonist] versus normalized response (three parameters) curve fit models were used for non‐linear regression of contractility data from cumulative exposure experiments; responses were normalized to spontaneous contractility prior to first treatment with inhibitor (Ami or CGS 15943; % MIT) or agonist (oxytocin; ΔMIT). Values for logIC_50_ and logEC_50_, respectively, were compared between groups within experiments using extra sum‐of‐squares *F* test.
