# Supplementary material for: Evaluating aminophylline and progesterone combination treatment to modulate contractility and labor‐related proteins in pregnant human myometrial tissues
Source: Pharmacol Res Perspect. 2021 Jul 5;9(4):e00818. doi: 10.1002/prp2.818 (PMC8256431; doi:10.1002/prp2.818)
Supplement: Supplementary file 1 — Fig S1‐S10 [file PRP2-9-e00818-s002.pdf]

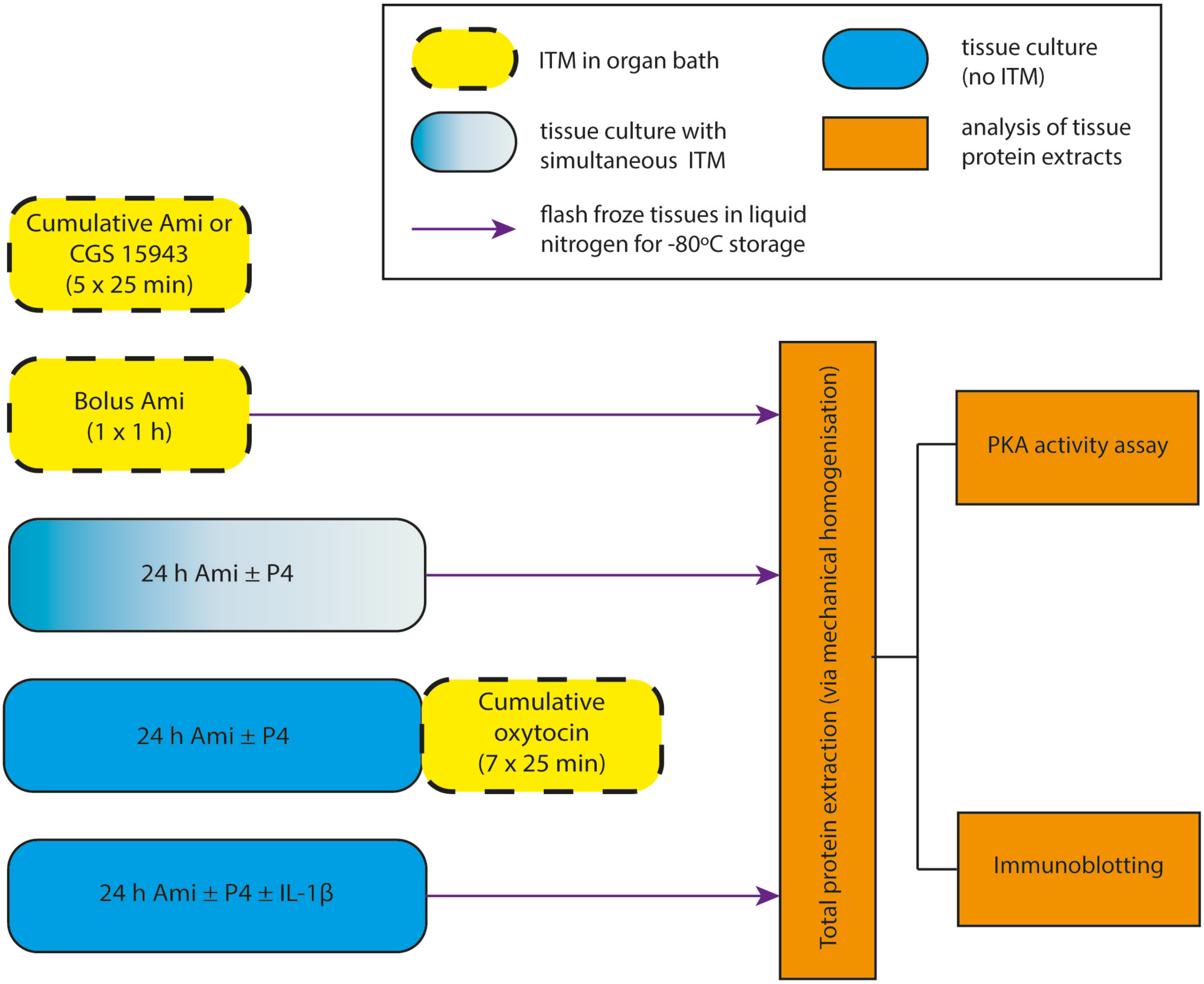

Figure S1. Workflow of experiments for data presented on assessing effects of aminophylline on contractility, PKA activity and protein abundance for progesterone receptors and contraction-associated proteins in *ex vivo* human myometrial tissues. Schematic diagram to show order of events (left to right) for experiments undertaken in the study, which are each described in the Methods of the main text. Abbreviations: isometric tension measurements (ITM), aminophylline (Ami), progesterone (P4), interleukin-1β (IL-1β), and protein kinase A (PKA).

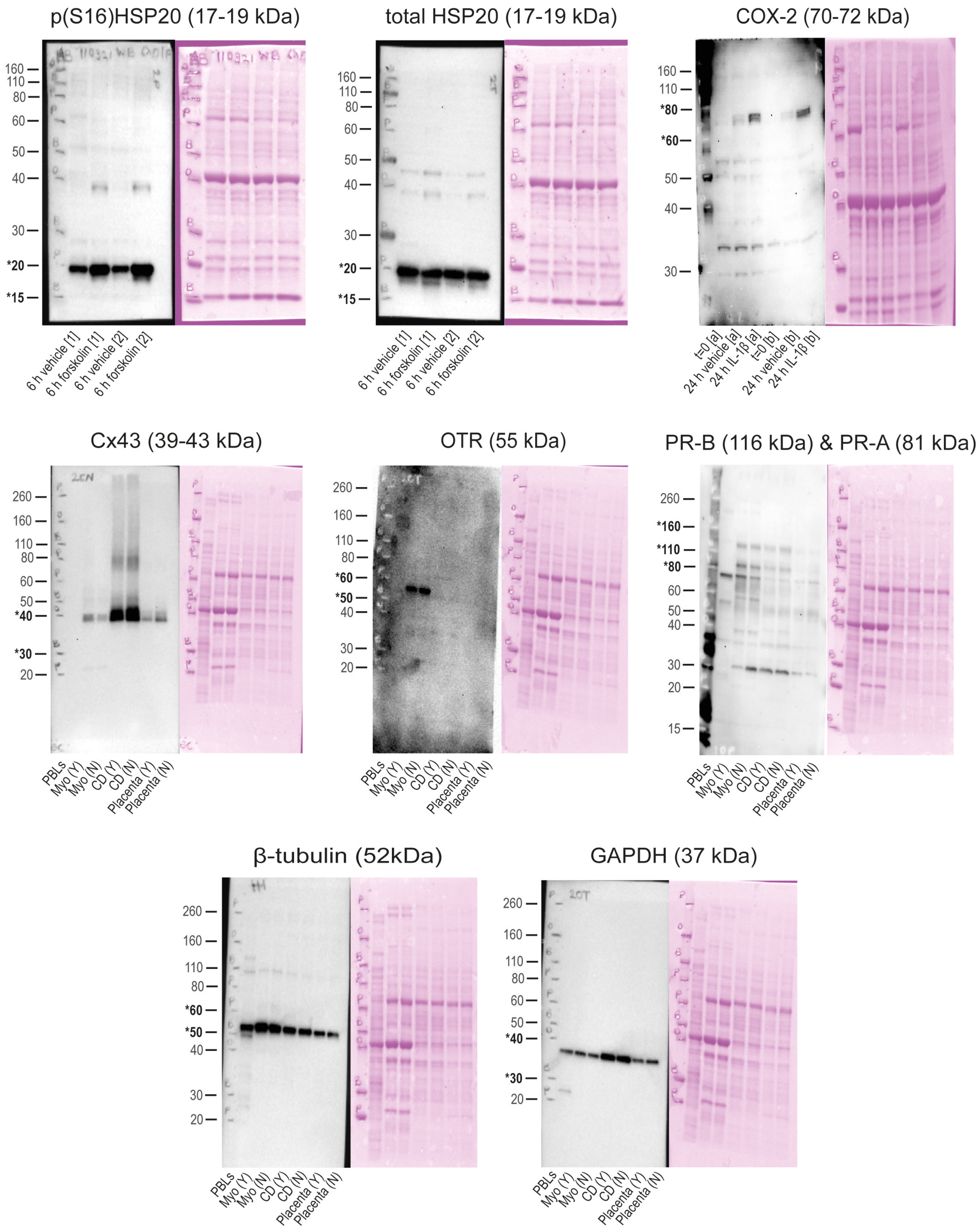

Figure S2. Representative images of Western blots for chemiluminescence and Ponceau S staining at validation of primary antibody specificities using total protein extracts of human reproductive tissues and peripheral blood leukocytes.

Figure S2. Representative images of Western blots for chemiluminescence and Ponceau S staining at validation of primary antibody specificities using total protein extracts of human reproductive tissues and peripheral blood leukocytes. Each primary antibody is represented by Western blot membrane-matched chemiluminescent (immunoblot; left) and pre-blocking Ponceau S stain (total protein; right) images. Molecular weight marker positions are annotated on the left of each chemiluminescent blot image, where markers in bold font (and each marked with an asterisk) indicate the region for detecting associated target protein (expected mass of which was determined using manufacturer-provided information and the UniProt knowledgebase (amino acid sequence and post-translational modifications considered); stated in headings). Ser16-phosphorylated ('p(16)') and total heat shock protein 20 (HSP20; PKA substrate) antibodies were used on extracts from four myometrial tissue strips from one biopsy, which had been incubated with forskolin (100 μM; positive control for HSP20 Ser16-phosphorylation) or its vehicle, without [1] or with [2] tension applied, during 6-h tissue culture. Cyclooxygenase-2 (COX-2) antibody was used on extracts from six myometrial tissue strips (three from each of two biopsies; [a] and [b]), which were untreated (t=0), or treated with interleukin-1β (1 ng/mL, positive control; IL-1β) or its vehicle for 24 h whilst under no tension. Antibodies for connexin-43 (Cx43), oxytocin receptor (OTR), progesterone receptor (isoforms A and B; PR-A and PR-B), β-tubulin and glyceraldehyde 3-phosphate dehydrogenase (GAPDH) were used on extracts from peripheral blood leukocytes (PBLs), along with untreated tissues dissected from biopsies of myometrium (Myo), choriodecidua (CD) and placenta. Samples from term pregnant women, who were non-labouring (N) or labouring (Y) at time of caesarean section; all consented using study ethics approval stated in Methods.

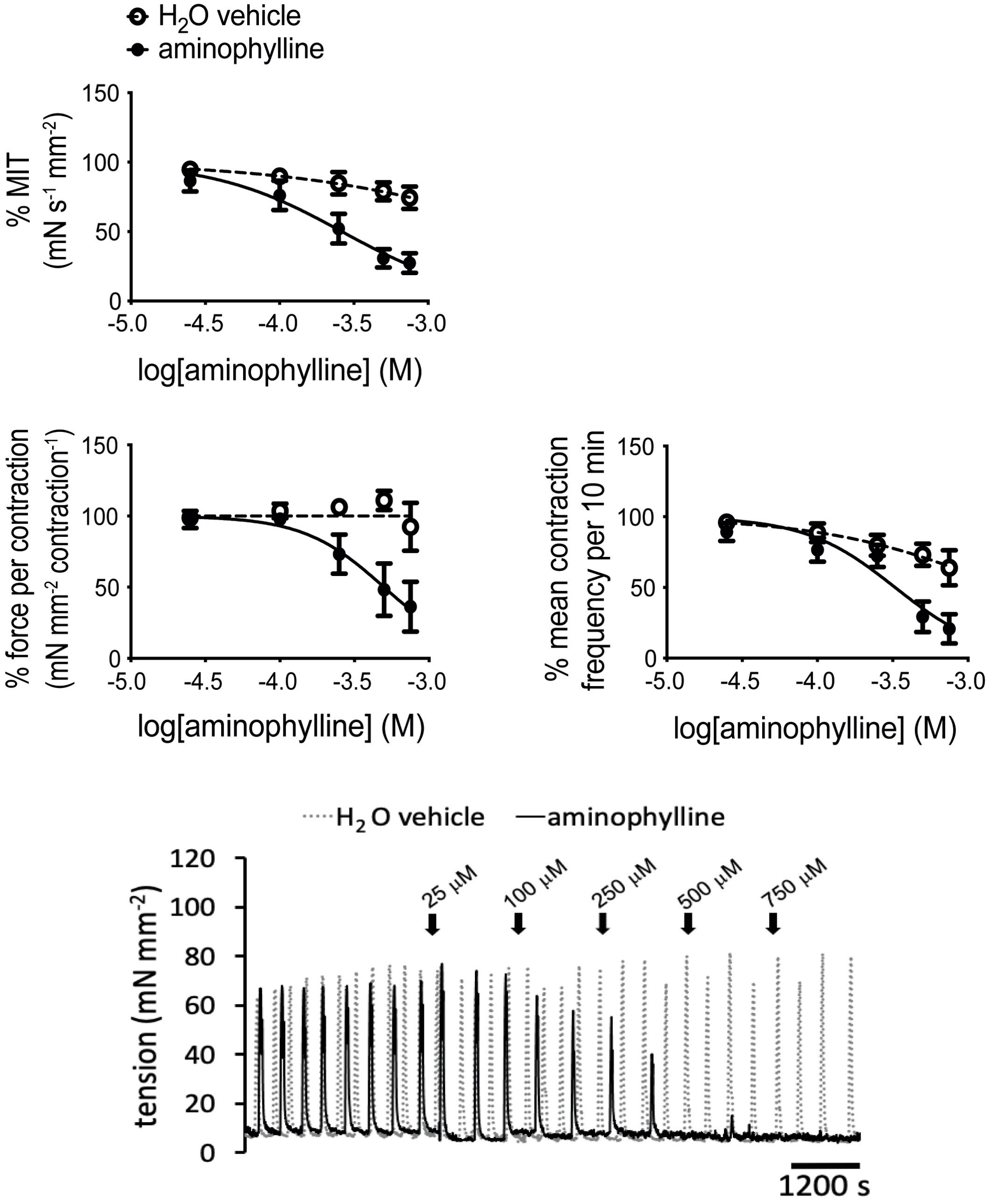

Figure S3. Cumulative concentration response to aminophylline from spontaneous contractions of human myometrial tissues. Isometric tension measurements obtained from myometrial tissues (biopsies from term pregnant non-labouring women; N=7), which were treated with aminophylline (25-750 μM) or its H_2O vehicle control in a cumulative manner (once every 25 min) starting from after establishing stable spontaneous contractions for ≥1 h. Data was analysed for mean integral tension (MIT), force per contraction and contraction frequency per 10 min to calculate % activity for each concentration of aminophylline (represented by the last 15-min of its treatment period) relative to the last 15-min of spontaneous contractions immediately before first application of aminophylline; all presented as mean ± SEM, where n=7 equates to number of biopsies for each dataset. Representative contractility profiles are shown for both aminophylline and its vehicle control from two tissue strips of one biopsy. Log[inhibitor] *vs* normalised response (variable slope) model was used for curve fitting by non-linear regression of all data. Extra sum-of-squares F test was used to compare log IC_{50} values for aminophylline *vs* its vehicle control from % MIT, $p<0.0001$.

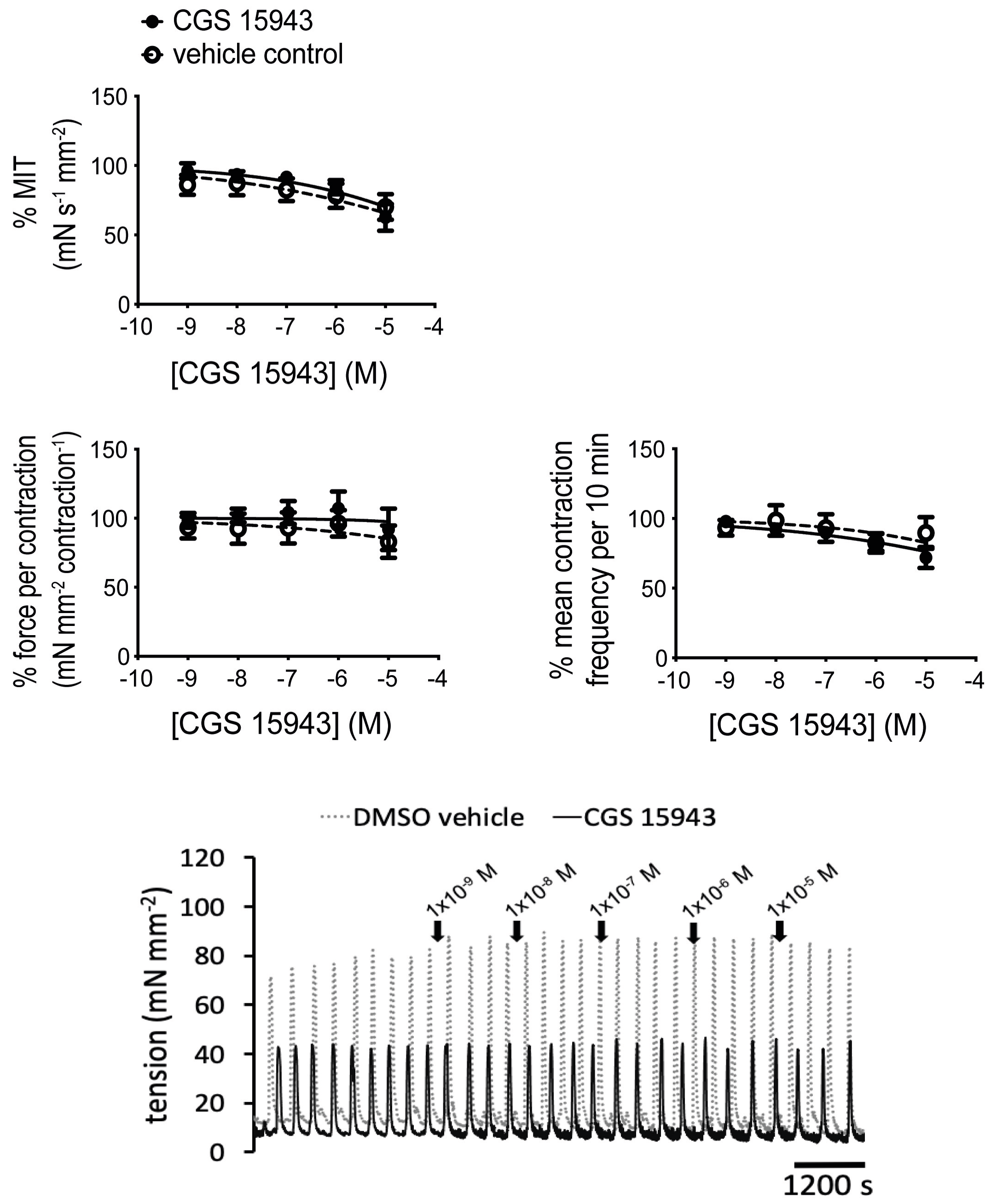

Figure S4. Cumulative concentration response to CGS 15943 from spontaneous contractions of human myometrial tissues. Isometric tension measurements obtained from myometrial tissues (biopsies from term pregnant non-labouring women; N=8), which were treated with either CGS 15943 (an adenosine receptor antagonist; $1x10^{-9}$ – $1x10^{-5}$ M) or its dimethyl sulfoxide (DMSO) vehicle control in a cumulative manner (once every 25 min) starting from after establishing stable spontaneous contractions for ≥1 h. Data was analysed for mean integral tension (MIT), force per contraction and contraction frequency per 10 min to calculate % activity for each concentration of CGS 15943 (represented by the last 15-min of its treatment period) relative to the last 15-min of spontaneous contractions immediately before first application of CGS 15943; all presented as mean ± SEM where n=8 equates to number of biopsies for each dataset. Representative contractility profiles are shown for both CGS 15943 and its vehicle control from biopsy-matched tissue strips. Log[inhibitor] *vs* normalised response (variable slope) model was used for curve fitting by non-linear regression of all data. Extra sum-of-squares F test was used to compare $logIC_{50}$ values between CGS 15943 and its vehicle control from % MIT, p=0.15.

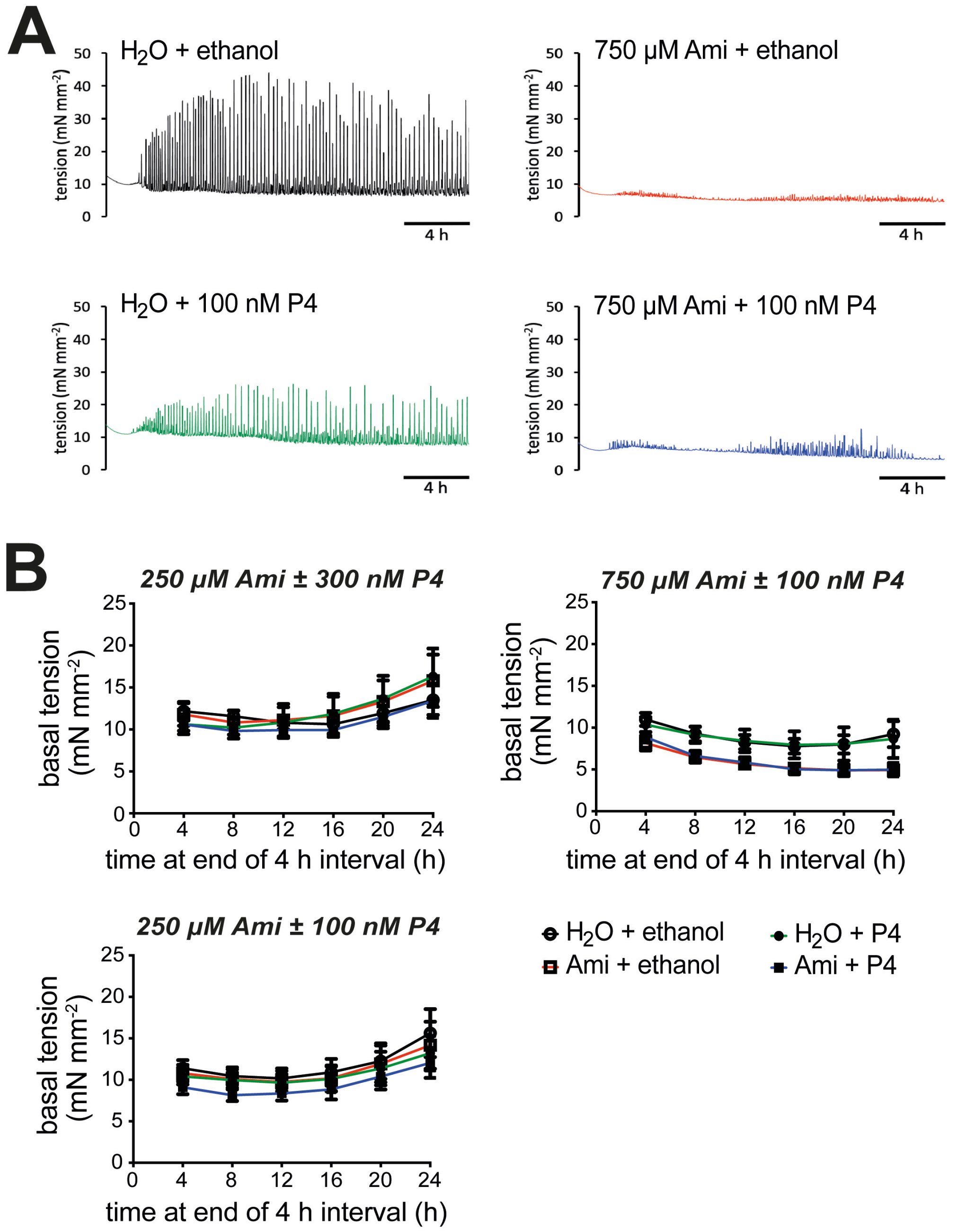

Figure S5. Basal tension of spontaneously contracting human myometrial tissues during 24-h aminophylline ± progesterone treatment. Isometric tension measurements of spontaneous contractions from myometrial tissues (biopsies from term pregnant non-labouring women; N=33) during 24-h treatment with combinations of aminophylline (Ami; H_2O vehicle) ± progesterone (P4; ethanol vehicle). Each biopsy (as a biological replicate, n) was dissected into four tissue strips and assigned to one of three sets of Ami±P4 treatments, namely 250 µM Ami ± 300 nM P4 (n=10), 750 µM Ami ± 100 nM P4 (n=11) and 250 µM Ami ± 100 nM P4 (n=12); these were added to serum-free culture media immediately after applying 29.4 mN tension at transducers. **(A)** Representative contractility profiles are shown for each Ami±P4 combination in a set of 750 µM Ami ± 100 nM P4 treatments for biopsy-matched tissue strips. **(B)** Data for basal tension at the end of every non-overlapping 4-h time 'interval' during each 24-h recording, which are presented as mean ± SEM for each Ami±P4 combination; times on x-axis are each relative to start of data recording immediately prior to applying 29.4 mN tension to tissues. Kruskal-Wallis test (Dunn's *post-hoc* analysis) was used for comparison of all Ami±P4 treatments at each time point of interest, and each of the three sets of Ami±P4 combinations were analysed separately; all $p>0.05$.

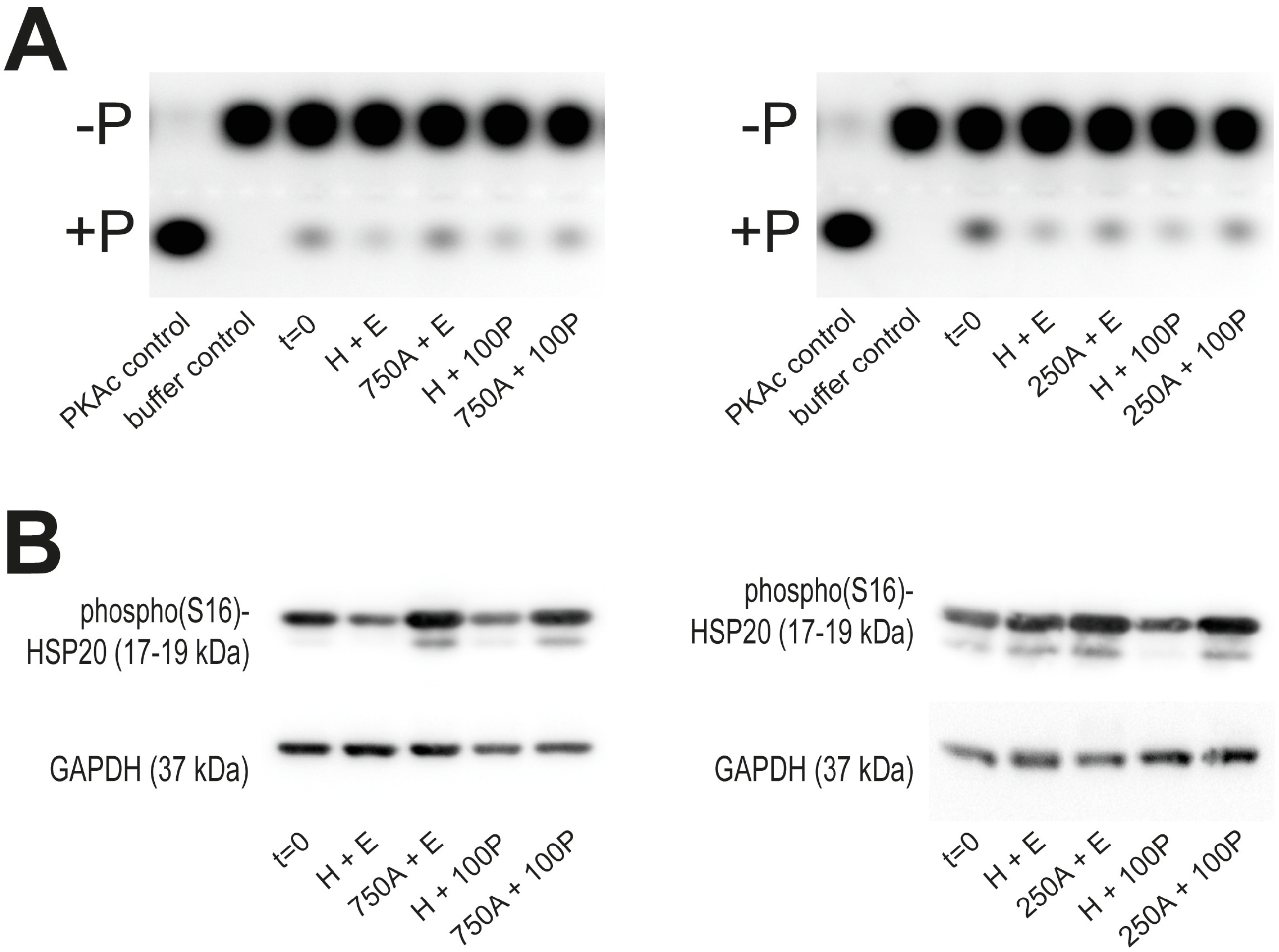

Figure S6. Representative images for PKA activity assay gels & Western blots for Ser16-phosphorylated HSP20 in human myometrial tissues after 24-h aminophylline ± progesterone treatment. Total protein extracts were prepared from tissue strips after isometric tension measurements, which were used to assess response at spontaneous contractions to combinations of aminophylline (250 (250A) or 750 (750A) µM; H_2O vehicle, H) ± progesterone (100 (100P) or 300 (300P) nM; ethanol vehicle, E) during 24-h treatment in tissue culture media (i.e. same tissues represented by Figure 3 & S5); 't=0' (i.e. untreated) biopsy-matched tissues were also extracted. These were used for PKA activity assays (purified catalytic PKA subunit (PKAc) used as a positive control and baseline background represented by 'buffer control'), and Western blotting for detection of Ser16-phosphorylated ('phospho(S16)') heat shock protein 20 (HSP20; a PKA substrate) along with glyceraldehyde 3-phosphate dehydrogenase (GAPDH; loading control). Representative images of **(A)** agarose gels for PKA assays, where '-P' and '+P' indicate non-phosphorylated and phosphorylated kemptide, respectively, along with **(B)** chemiluminescent Western blots, are shown for 750A ± 100P (left panel) and 250A ± 100P (right panel) treatments applied to biopsy-matched tissue strips; their associated data histograms are presented at Figure 4.

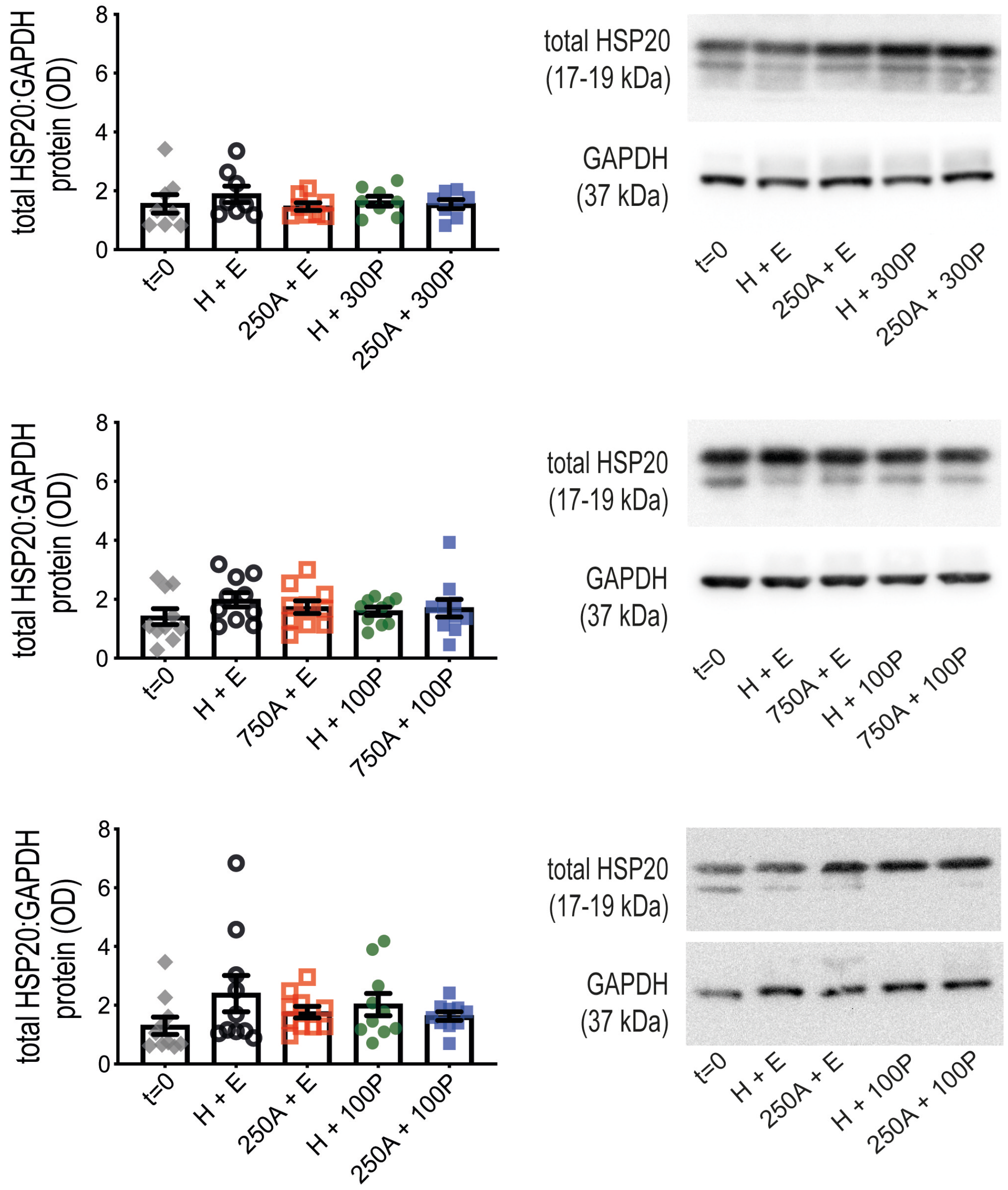

Figure S7. Protein abundance for total HSP20 in human myometrial tissues after 24-h aminophylline ± progesterone treatment. Total protein extracts were prepared from tissue strips after isometric tension measurements, which were used to assess response at spontaneous contractions to combinations of aminophylline (250 (250A) or 750 (750A) μM; H_2O vehicle, H) ± progesterone (100 (100P) or 300 (300P) nM; ethanol vehicle, E) during 24-h treatment in tissue culture media (i.e. same tissues represented by Figure 3 & S5); 't=0' (i.e. untreated) biopsy-matched tissues were also extracted. These were used for detection by Western blotting of total heat shock protein 20 (HSP20; a PKA substrate) and glyceraldehyde 3-phosphate dehydrogenase (GAPDH; loading control). Data presented as mean ± SEM for 250A ± 300P (n=8), 750A ± 100P (n=10) and 250A ± 100P (n=10); n equates to number of biopsies for each dataset. Representative images of chemiluminescent Western blots shown adjacent to their associated histograms. Repeated measures ANOVA with Geisser-Greenhouse correction (Dunnett's or Tukey's *post-hoc* analysis) or Friedman test (Dunn's *post-hoc* analysis) was used for (i) t=0 *vs* each 24-h treatment (all $p>0.05$), and (ii) comparisons between 24-h treatments only (all $p>0.05$).

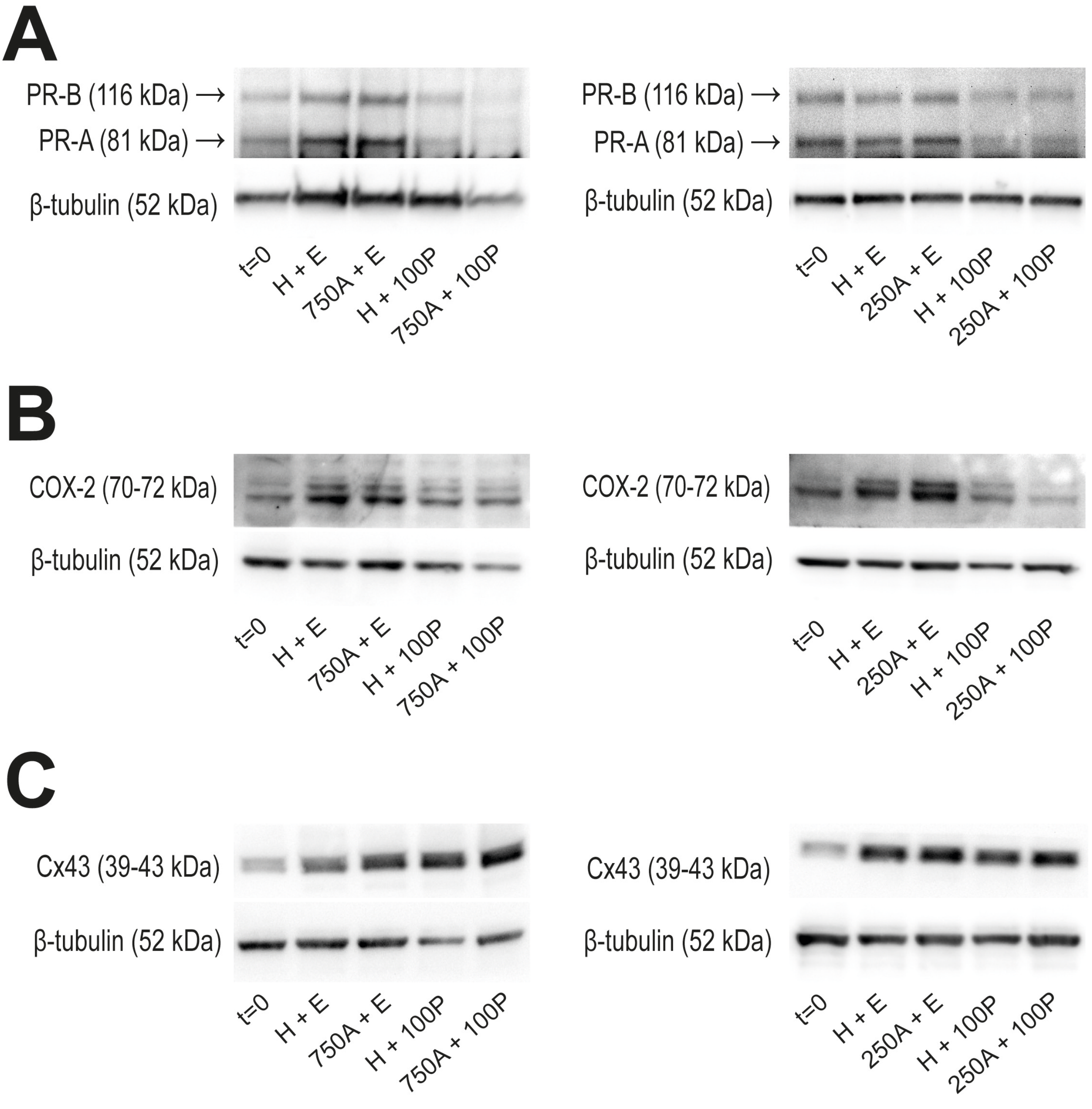

Figure S8. Representative Western blot images for PR, COX-2 and Cx43 in human myometrial tissues after 24-h aminophylline ± progesterone treatment. Total protein extracts were prepared from tissue strips after isometric tension measurements, which were used to assess response at spontaneous contractions to combinations of aminophylline (250 (250A) or 750 (750A) µM; H_2O vehicle, H) ± progesterone (100 (100P) or 300 (300P) nM; ethanol vehicle, E) during 24-h treatment in tissue culture media (i.e. same tissues represented by Figure 3 & S5); 't=0' (i.e. untreated) biopsy-matched tissues were also extracted. These were used for detection by Western blotting of **(A)** progesterone receptor (PR), both isoforms A (PR-A) and B (PR-B), **(B)** cyclooxygenase-2 (COX-2), and **(C)** connexin-43 (Cx43), all along with β-tubulin (loading control). Representative images of chemiluminescent Western blots are shown for 750A ± 100P (left panel) and 250A ± 100P (right panel) treatments applied to biopsy-matched tissue strips; their associated data histograms are presented at Figures 5-7.

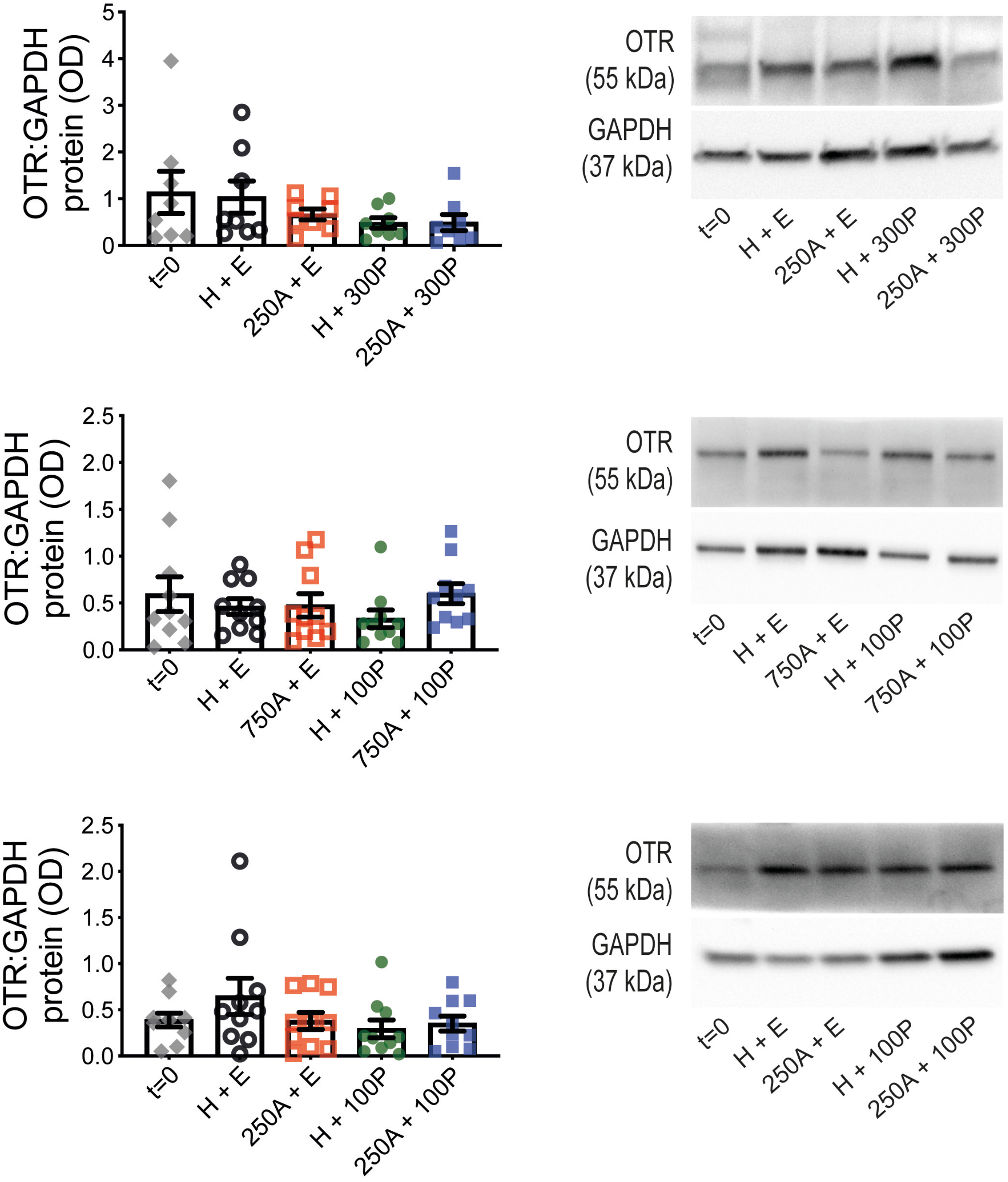

Figure S9. Protein abundance for OTR in human myometrial tissues after 24-h aminophylline ± progesterone treatment. Total protein extracts were prepared from tissue strips after isometric tension measurements, which were used to assess response at spontaneous contractions to combinations of aminophylline (250 (250A) or 750 (750A) µM; H_2O vehicle, H) ± progesterone (100 (100P) or 300 (300P) nM; ethanol vehicle, E) during 24-h treatment in tissue culture media (i.e. same tissues represented by Figure 3); ‘t=0’ (i.e. untreated) biopsy-matched tissues were also extracted. These were used for detection by Western blotting of oxytocin receptor (OTR) and glyceraldehyde 3-phosphate dehydrogenase (GAPDH; loading control). Data presented as mean ± SEM for 250A ± 300P (n=8), 750A ± 100P (n=10) and 250A ± 100P (n=10); n equates to number of biopsies for each dataset. Representative images of chemiluminescent Western blots shown adjacent to their associated histograms. Friedman test (Dunn’s *post-hoc* analysis) was used for (i) t=0 *vs* each 24-h treatment, and (ii) comparisons between 24-h treatments only; all $p>0.05$.

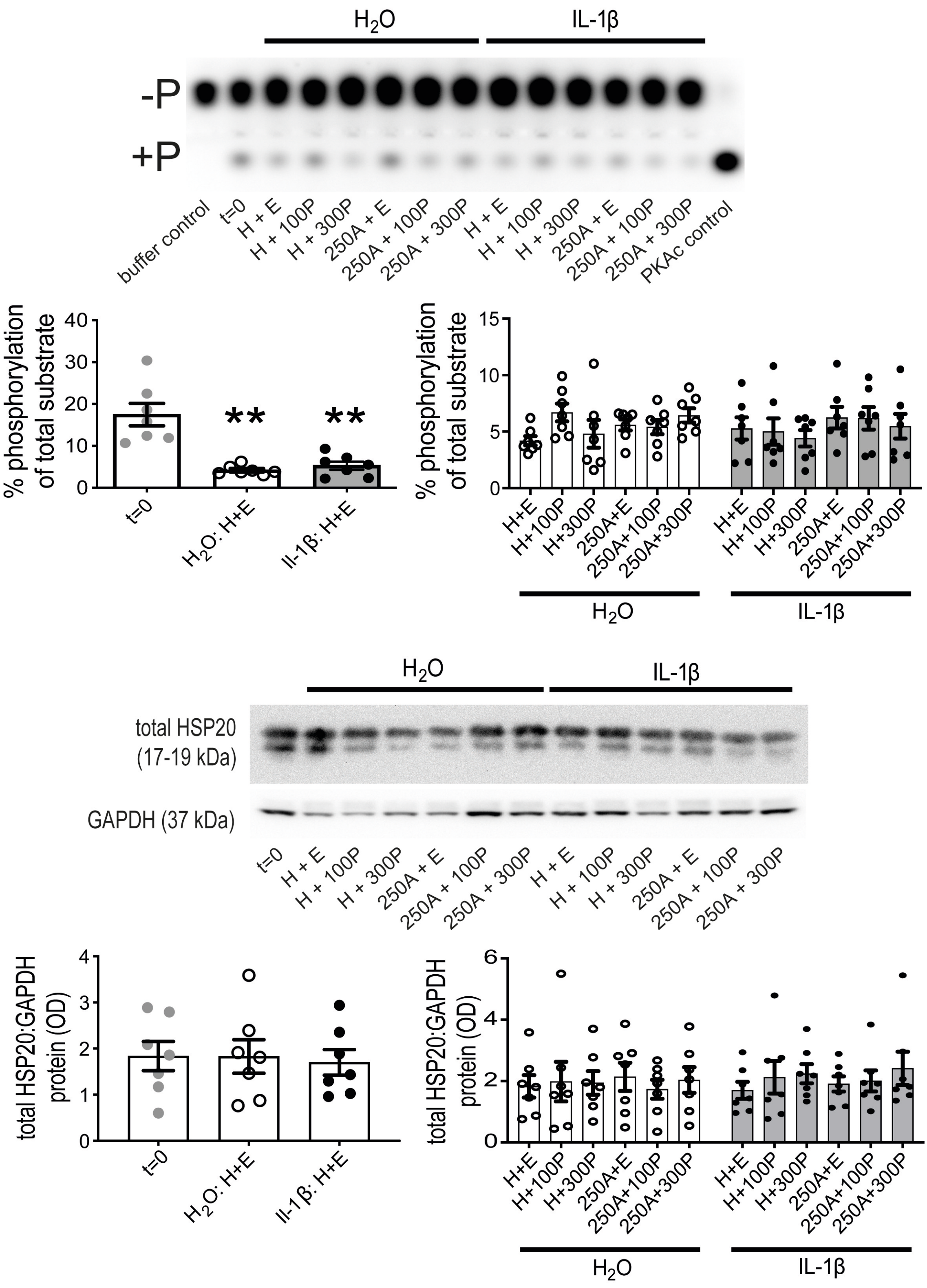

Figure S10. Total PKA activity & HSP20 protein abundance in human myometrial tissues after 24-h aminophylline ± progesterone ± IL-1β treatment.

Figure S10. Total PKA activity & HSP20 protein abundance in human myometrial tissues after 24-h aminophylline ± progesterone ± IL-1β treatment. Total protein extracts were prepared from tissue strips after treatment in serum-free culture media with combinations of aminophylline (250 µM (250A); H_2O vehicle, H) ± progesterone (100 (100P) or 300 (300P) nM; ethanol vehicle, E) ± interleukin-1β (1 ng/mL in H_2O vehicle; IL-1β) for 24 h while maintained under isotonic tension (~4 mN); 't=0' (i.e. untreated) biopsy-matched tissues were also extracted. These were used for PKA activity assays (purified catalytic PKA subunit (PKAc) used as a positive control and baseline background represented by 'buffer control') and Western blotting of total heat shock protein 20 (HSP20, a PKA substrate) along with glyceraldehyde 3-phosphate dehydrogenase (GAPDH; loading control). Representative images of agarose gels for PKA assays (top panel), where '-P' and '+P' indicate non-phosphorylated and phosphorylated kemptide, respectively, and chemiluminescent Western blots (bottom panel) are shown above their associated histograms. All data presented as mean ± SEM (n=7); n equates to number of biopsies for each dataset. In both cases, (i) repeated measures ANOVA with Geisser-Greenhouse correction (Dunnett's *post-hoc* analysis) was used for t=0 *vs* each 24-h treatment (** $p \leq 0.01$), and (ii) repeated measures ANOVA with Geisser-Greenhouse correction (Tukey's *post-hoc* analysis) or Friedman test (Dunn's *post-hoc* analysis) was used for comparisons between 24-h treatments only (IL-1β and its vehicle control analysed separately; all $p > 0.05$); two-way repeated measures ANOVA (Sidak's *post-hoc* analysis) was used for IL-1β *vs* its vehicle control comparison for matched A±P treatments (all $p > 0.05$).

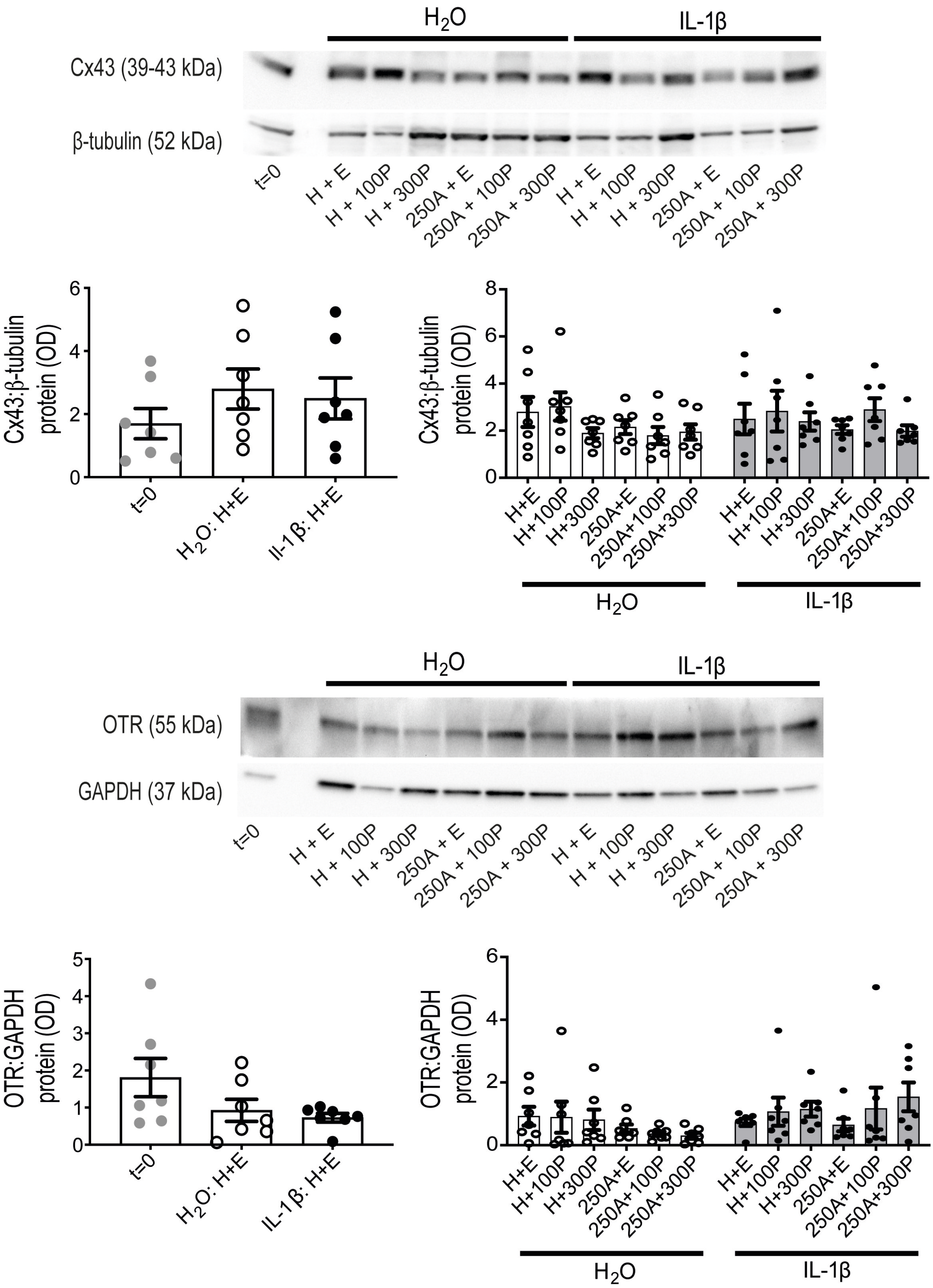

Figure S11. Protein abundance for Cx43 & OTR in human myometrial tissues after 24-h aminophylline ± progesterone ± IL-1β treatment.

Figure S11. Protein abundance for Cx43 & OTR in human myometrial tissues after 24-h aminophylline ± progesterone ± IL-1β treatment. Total protein extracts were prepared from tissue strips after treatment in serum-free culture media with combinations of aminophylline (250 μM (250A); H_2O vehicle, H) ± progesterone (100 (100P) or 300 (300P) nM; ethanol vehicle, E) ± interleukin-1β (1 ng/mL in H_2O vehicle; IL-1β) for 24 h while maintained under isotonic tension (~4 mN); 't=0' (i.e. untreated) biopsy-matched tissues were also extracted. These were used for detection by Western blotting of connexin-43 (Cx43; top panel) and oxytocin receptor (OTR; bottom panel), along with glyceraldehyde 3-phosphate dehydrogenase (GAPDH) and β-tubulin (loading controls). Representative images of chemiluminescent Western blots are shown for biopsy-matched tissue strips above their associated histograms. All data presented as mean ± SEM (n=7); n equates to number of biopsies for each dataset. In both cases, (i) repeated measures ANOVA with Geisser-Greenhouse correction (Dunnett's *post-hoc* analysis) was used for t=0 *vs* each 24-h treatment (all $p>0.05$), and (ii) repeated measures ANOVA with Geisser-Greenhouse correction (Tukey's *post-hoc* analysis) or Friedman test (Dunn's *post-hoc* analysis) was used for comparisons between 24-h treatments only (IL-1β and its vehicle control analysed separately; all $p>0.05$); two-way repeated measures ANOVA (Sidak's *post-hoc* analysis) was used for IL-1β *vs* its vehicle control comparison for matched A ± P treatments (all $p>0.05$).
