# Supplementary material for: Evaluating aminophylline and progesterone combination treatment to modulate contractility and labor‐related proteins in pregnant human myometrial tissues
Source: Pharmacol Res Perspect. 2021 Jul 5;9(4):e00818. doi: 10.1002/prp2.818 (PMC8256431; doi:10.1002/prp2.818)
Supplement: Supplementary file 2 — Table S1‐S5 [file PRP2-9-e00818-s001.docx]

**Supporting Information - Table S1: Antibodies for Immunoblotting**

|  | Antibody target | Supplier (catalog no.) | Antibody species | Working  dilution | Molecular weight of target protein (kDa) | RRID |
| --- | --- | --- | --- | --- | --- | --- |
| *Primary antibodies* | Ser16-phosphorylated heat shock protein 20 (HSP20)† | Abcam (ab58522) | Polyclonal rabbit | 1:10000^a^ | 17-19 | AB_883036 |
|  | Total HSP20§^b^ | Abcam (ab184161) | Monoclonal (EPR14458) rabbit | 1:80000^aa^ | 17-19 | AB_2833086 |
|  | Cyclooxygenase-2 (COX-2)† | Santa Cruz Biotechnology  (sc-1745) | Polyclonal goat | 1:1000 | 70-72 | AB_631309 |
|  | Oxytocin receptor (OTR)‡ | Santa Cruz Biotechnology  (sc-8102) | Polyclonal goat | 1:1000 | 55 | AB_2157765 |
|  | Connexin-43 (Cx43)‡ | Cell Signalling Technology (3512) | Polyclonal rabbit | 1:1000 | 39 | AB_2294590 |
|  | Progesterone receptor (PR; both isoforms A and B)‡ | Santa Cruz Biotechnology  (sc-7208) | Polyclonal goat | 1:400 | PR-B: 116  PR-A: 81 | AB_2164331 |
|  | Glyceraldehyde 3-phosphate dehydrogenase (GAPDH)§^bb^ | Millipore (MAB374) | Monoclonal (6C5) mouse | 1:40000^aa^ | 37 | AB_2107445 |
|  | β-tubulin§ | Abcam (ab21057) | Polyclonal goat | 1:2000 | 52 | AB_727043 |
| *Secondary antibodies (all HRP-conjugated)* | Rabbit IgG§ | Cell Signalling Technology (7074) | Polyclonal goat | 1:2000^b^ | n/a | AB_2099233 |
|  | Goat Ig (all classes)§ | Dako/Agilent (P0449) | Polyclonal rabbit | 1:2000 | n/a | AB_2617143 |
|  | Mouse IgG§ | Cell Signalling Technology  (7076) | Polyclonal horse | 1:2000^bb^ | n/a | AB_330924 |

*Abbreviations: RRID – Research Resource Identifier, Ig – immunoglobulins, HRP – horse radish peroxidase;* diluted antibodies † not re-used, ‡ re-used once, or § re-used twice; antibodies diluted in Tris-buffered saline with Tween-20 ± blocking agent (^a^5 % w/v bovine serum albumin or ^aa^5% fat-free milk); secondary antibodies diluted ^b^1:8000 and ^bb^1:4000 for specifically associated primary antibodies. Figure S2 provides representative images of Western blots for validation of antibody specificity using human myometrium, choriodecidua, placenta and leukocytes.

**Supporting Information - Table S2: Patient Demographics for Myometrium Biopsies – Grouped by Type of Experiment**

|  | Acute cumulative Ami and/or CGS 15943 response (N=13)^†^ | Acute (1 h) bolus Ami response (N=10)^‡^ | 24 h Ami ± P4 treatment during ITM in TC (N=33)^§^ | 24 h Ami ± P4 TC for ITM with cumulative oxytocin stimulation (N=9)^§^ | 24 h Ami ± P4 ± IL-1β TC without ITM (N=7)^§^ |
| --- | --- | --- | --- | --- | --- |
| *Maternal age (years; median with range)* | 36 (22 to 43) | 36 (31 to 40) | 35 (26 to 48) | 38 (33 to 47) | 35 (28 to 48) |
| *Gestational age (weeks^+days^; median with range)* | 39^+0^ (37^+6^ to 40^+5^) | 39^+0^ (37^+1^ to 40^+0^) | 39^+2^ (37^+3^ to 40^+1^) | 39^+2^ (38^+1^ to 40^+2^) | 39^+0^ (38^+3^ to 40^+5^) |
| *Gravida (median with range)* | 2 (1 to 5) | 2 (1 to 3) | 2 (1 to 4) | 2 (1 to 3) | 3 (1 to 6) |
| *Parity (median with range)* | Viable = 1 (0 to 2)  Non-viable & abortus = 0 (0 to 2) | Viable = 1 (0 to 1)  Non-viable & abortus = 1 (0 to 1) | Viable = 0 (0 to 1)  Non-viable & abortus = 0 (0 to 3) | Viable = 1 (0 to 2)  Non-viable & abortus = 0 (0 to 2) | Viable = 1 (0 to 2)  Non-viable & abortus = 0 (0 to 5) |
| *Booking BMI*  *(median with range)* | 22 (18 to 27) | 22 (17 to 29) | 22 (18 to 37) | 21 (20 to 23) | 22 (19 to 32) |
| *Ethnicity (as self-specified by participant)* | White European = 6 British, 3 unspecified;  White other = 1 North American (USA);  Asian = 1 Indian;  Stated as ‘other’ = 1 unspecified;  Stated as ‘mixed’ = 1 unspecified | White European = 2 British, 1 German-Italian, 1 Spanish,  2 Polish;  White other = 1 Australian, 1 South African;  Asian = 1 Indian;  Not stated = 1 | White European = 10 British, 2 Bulgarian, 2 Czech, 1 Irish, 1 Portuguese, 2 Serbian, 1 Swedish;  White other = 1 Argentinian, 1 Brazilian, 2 South African, 1 New Zealander;  Black African/Caribbean = 1 unspecified;  Asian = 1 Filipino, 1 Indian;  Stated as ‘other’ = 1 Brazilian, 2 Iranian;  Stated as ‘mixed’ = 1 White British-South African, 1 White South African-Black South African, 1 unspecified | White European = 3 British, 1 Cypriot;  White other = 1 Brazilian-Italian, 1 New Zealander;  Asian = 1 Thai;  Stated as ‘other’ = 1 Iranian;  Stated as ‘mixed’ = 1 unspecified | White European = 2 British, 1 Czech,  1 Irish;  White other = 1 South African;  Stated as ‘mixed’ = 1 British-Iranian, 1 unspecified |
| *Reason for caesarean^¶^* | Previous caesarean = 6  Breech = 3  Other indications = 4 | Previous caesarean = 6  Breech = 2  Other indications = 2 | Previous caesarean = 14  Breech = 7  Maternal request = 4  Other indications = 8 | Previous caesarean = 2  Maternal request = 4  Other indications = 3 | Previous caesarean = 4  Maternal request = 1  Other indications = 2 |

*Abbreviations: Ami, aminophylline; P4, progesterone; ITM, isometric tension measurements; TC, tissue culture; IL-1β, interleukin-1β; BMI, body mass index.*

N denotes number of biopsies from unique participants, where some provided enough tissue strips to use for more than one of set of experimental conditions and direct comparisons between their datasets were avoided:

**^†^** For cumulative stimulation, two biopsies were dissected and shared across two different treatments with vehicle controls (Ami and CGS 15943); the rest of the biopsies were each used for either Ami or CGS 15943 (with their vehicle controls) but not both.

**^‡^** For 1 h bolus stimulation, six biopsies were dissected and shared across three Ami concentrations of interest (100, 250 and 750 μM) for 1 h bolus response, whereas two biopsies were shared for two Ami concentrations and two biopsies were assigned to one Ami concentration each due to lower number of tissue strips with stable baseline (i.e. before Ami treatment) spontaneous contractions.

**^§^** Some biopsies were shared between (i) 24 h spontaneous contractility measurements in the presence of Ami ± P4 during TC, and (ii) 24 h Ami ± P4 (± IL-1β) treatment during TC with (three biopsies) or without (two biopsies) subsequent measurements for oxytocin-stimulated contractions.

**^¶^** Reasons for caesarean identified as ‘other indications’ included maternal previous third- or fourth- degree tear (N=3), placenta previa (N=2), previous miscarriage/stillbirth (N=1), previous shoulder dystocia (N=1), previous rectal prolapse (N=1), large for gestational age fetal growth (N=2), neonatal cardiac condition (N=2), congenital dysplastic hip (N=1), maternal age (N=4), maternal lymphangioma of the left orbit (N=1), maternal congenital heart condition (N=1); no more than two of each condition was represented for each of the five groups of samples tabulated. Exclusion criteria for singleton pregnant women: diagnosis of diabetes, pre-eclampsia, obstetric cholestasis or blood-borne infections, along with prescribed recent use of vasodilators or bronchodilators.

**Supporting Information - Table S3: Patient Demographics for Myometrium Biopsies – Grouped by Ami ± P4 Treatment during 24 h ITM in TC**

|  | 250 μM Ami ± 300 nM P4 (n=10) | 750 μM Ami ± 100 nM P4 (n=11) | 250 μM Ami ± 100 nM P4 (n=12) |
| --- | --- | --- | --- |
| *Maternal age (years; median with range)* | 36 (32 to 48) | 34 (26 to 37) | 36 (29 to 42) |
| *Gestational age (weeks^+days^; median with range)* | 39^+0^ (38^+3^ to 39^+5^) | 39^+1^ (37^+3^ to 40^+0^) | 39^+3^ (39^+0^ to 40^+1^) |
| *Gravida (median with range)* | 2 (1 to 4) | 2 (1 to 3) | 2 (1 to 3) |
| *Parity (median with range)* | Viable = 0 (0 to 1)  Non-viable & abortus = 0 (0 to 3 | Viable = 0 (0 to 1)  Non-viable & abortus = 0 (0 to 1) | Viable = 1 (0 to 1)  Non-viable & abortus = 0 (0 to 1) |
| *Booking BMI*  *(median with range)* | 22 (19 to 30) | 20 (18 to 28) | 23 (18 to 37) |
| *Ethnicity (as self-specified by participant)* | White European = 2 British, 1 Czech;  White other = 1 Argentinian, 1 South African, 1 New Zealander;  Asian = 1 Indian;  Stated as ‘other’ = 2 Iranian;  Stated as ‘mixed’ =  1 White British-South African | White European = 3 British, 1 Bulgarian, 1 Czech, 2 Serbian, 1 Swedish;  White other = 1 Brazilian, 1 South African;  Black African = 1 unspecified | White European = 5 British, 1 Bulgarian, 1 Irish, 1 Portuguese  Asian = 1 Filipino;  Stated as ‘other’ = 1 Brazilian;  Stated as ‘mixed’ = 1 White South African-Black South African, 1 unspecified |
| *Reason for caesarean^†^* | Previous caesarean = 4  Breech = 1  Maternal request = 1  Other indications = 4 | Previous caesarean = 5  Breech = 3  Maternal request = 1  Other indications = 2 | Previous caesarean = 5  Breech = 3  Maternal request = 2  Other indications = 2 |

*Abbreviations: ITM, isometric tension measurements; TC, tissue culture; Ami, aminophylline; P4, progesterone; BMI, body mass index.* Kruskal-Wallis (Dunn’s *post-hoc*) test used to compare all three sets of Ami ± P4 treatment conditions showed no statistically significant differences (p>0.05) for each parameter.

**^†^** Reasons for caesarean identified as ‘other indications’ included maternal previous fourth-degree tear (N=1), placenta previa (N=1), large for gestational age fetal growth (N=2), neonatal cardiac condition (N=1), maternal age (N=1), maternal lymphangioma of the left orbit (N=1), maternal congenital heart condition (N=1); no more than two of each condition was represented for each of the three groups of samples tabulated. Exclusion criteria for singleton pregnant women: diagnosis of diabetes, pre-eclampsia, obstetric cholestasis or blood-borne infections, along with prescribed recent use of vasodilators or bronchodilators.

**Supporting Information - Table S4: Dimensions, Weights & Tensions (Mean ± SEM) of Myometrial Tissue Strips – Acute Cumulative & Bolus Vehicle Control Treatments at ITM**

|  | **Acute cumulative Ami vehicle at spontaneous contractions (n=7)^†^** | **Acute cumulative CGS 15943 vehicle at spontaneous contractions (n=6)^†^** | **Acute (1 h) bolus Ami vehicle at spontaneous contractions (n=8)^‡^** |
| --- | --- | --- | --- |
| No tension length before setting to ITM slack length (mm) | 9.1 ± 0.40 | 8.7 ± 0.61 | 10.0 ± 0.57 |
| No tension CSA before setting to ITM slack length (mm^2^) | 0.6 ± 0.08 | 0.6 ± 0.07 | 0.9 ± 0.07 |
| ITM slack length (mm) | 10.0 ± 0.53 | 10.0 ± 0.63 | 10.9 ± 0.44 |
| CSA at ITM slack length (mm^2^) | 0.6 ± 0.09 | 0.5 ± 0.07 | 0.8 ± 0.06 |
| Length added by applying 29.4 mN tension (ΔL; mm) | 4.9 ± 0.54 | 5.4 ± 0.64 | 6.2 ± 0.48 |
| Strain (ΔL/L; 29.4 mN tension applied vs. ITM slack length) | 0.5 ± 0.05 | 0.5 ± 0.05 | 0.6 ± 0.03 |
| CSA after applying 29.4 mN tension (mm^2^) | 0.4 ± 0.06 | 0.3 ± 0.04 | 0.5 ± 0.04 |
| Tissue weight (mg) | 60.1 ± 7.39 | 56.4 ± 10.02 | 94.1 ± 8.83 |

*Abbreviations: ITM, isometric tension measurements; CSA, cross-sectional area; Ami, aminophylline.* All data from vehicle control tissues. Biopsies are the same as those represented by Table S2. Mann-Whitney tests or Welch’s t-tests for each parameter at Ami *vs* H_2_O and CGS 15943 *vs* DMSO comparisons showed no statistically significant differences (*p*>0.05).

**^†^** N=2 biopsies were dissected and shared across two different treatments with vehicle controls (Ami and CGS 15943); the rest of the biopsies were each used for either Ami or CGS 15943 (with their vehicle controls) but not both.

**^‡^** Data from vehicle controls represented by 1 h bolus 250 μM Ami experiments only.

**Supporting Information - Table S5: Dimensions, Weights & Tensions (Mean ± SEM) of Myometrial Tissue Strips – 24 h TC ± ITM for Vehicle Controls**

|  | **24 h ITM in TC: 750 μM Ami ± 100 nM P4 (n=8)** | **24 h ITM in TC: 250 μM Ami ± 100 nM P4 (n=10)^†^** | **24 h ITM in TC: 250 μM Ami ± 300 nM P4 (n=8)^†^** | **24 h Ami ± P4 TC for ITM with oxytocin (n=8)** | **24 h TC only:**  **Ami ± P4 ± IL-1β (n=7)** |
| --- | --- | --- | --- | --- | --- |
| t=0 (no tension) length (mm)**^‡^** | 11.6 ± 0.26 | 11.0 ± 0.52 | 10.9 ± 0.52 | 9.8 ± 0.31 | Not recorded |
| t=0 (no tension) CSA (mm^2^) | 0.7 ± 0.06 | 0.7 ± 0.05 | 0.7 ± 0.04 | 0.7 ± 0.06 | Not recorded |
| Isotonic force applied during TC (mN) | n/a | n/a | n/a | 3.9 ± 0.05 | 4.0 ± 0.05 |
| Tissue length after 24 h TC + isotonic force (mm) | n/a | n/a | n/a | 8.9 ± 0.55 | 10.0 ± 0.85 |
| Length added by isotonic force during 24 h TC relative to t=0 (ΔL; mm) | n/a | n/a | n/a | -0.87 ± 0.48 | n/a |
| Strain (ΔL/L; 24 h TC + isotonic force vs. t=0) | n/a | n/a | n/a | -0.1 ± 0.05 | n/a |
| Stress from 24 h isotonic force (mN mm^-2^)**^§^** | n/a | n/a | n/a | 5.9 ± 1.03 | 5.2 ± 0.53 |
| CSA after 24 h TC + isotonic force (mm^2^) | n/a | n/a | n/a | 0.7 ± 0.09 | 0.8 ± 0.09 |
| ITM slack length (mm) | Not recorded | Not recorded | Not recorded | 9.4 ± 0.57 | n/a |
| CSA at ITM slack length (mm^2^) | n/a | n/a | n/a | 0.7 ± 0.08 | n/a |
| Length added by manual stretch at ITM relative to t=0 or slack length (ΔL; mm)**^¶^** | 6.8 ± 0.56 | 6.4 ± 0.44 | 6.5 ± 0.61 | 4.7 ± 0.28 | n/a |
| Strain (ΔL/L; manual stretch at ITM vs. t=0 or ITM slack length)**^¶^** | 0.6 ± 0.04 | 0.6 ± 0.06 | 0.6 ± 0.05 | Set to 0.5 for 1.5 x slack length stretch | n/a |
| Peak tension from manual stretch of tissues to 1.5 x slack length at ITM (mN) | n/a | n/a | n/a | 33.9 ± 6.62 | n/a |
| CSA from tissue length measured at end of ITM or sum of ‘ΔL/L + ITM slack length’ (mm^2^) | 0.6 ± 0.04 | 0.6 ± 0.05 | 0.6 ± 0.04 | 0.5 ± 0.06 | n/a |
| Tissue weight (mg) | 89.0 ± 8.37 | 77.7 ± 6.53 | 77.8 ± 5.07 | 67.0 ± 6.17 | 84.0 ± 9.5 |

*Abbreviations: TC, tissue culture; ITM, isometric tension measurements; CSA, cross-sectional area; Ami, aminophylline; P4, progesterone; IL-1β, interleukin-1β.* All data from vehicle control tissues; biopsies for which some tissue strip or micrometer measurements were mistakenly not documented during 24 h ITM in TC experiments have not been included. Biopsies are the same as those represented by Tables S2 and S3. Kruskal-Wallis (Dunn’s *post-hoc*) or Brown-Forsythe & Welch ANOVA (Dunnett’s T3 *post-hoc*) tests for each parameter at all treatment pairings for each set of experiments showed no differences (p>0.05); two-way ANOVA with Bonferroni’s *post-hoc* analysis for IL-1β *vs* its H_2_O vehicle also showed no statistically significant differences associated with IL-1β for all parameters at ‘24 h TC only’ experiments.

**^†^** Some biopsies were each dissected into two separate sets of tissue strips to use for (i) 24 h spontaneous contractility measurements in the presence of Ami ± P4 during TC, and (ii) 24 h Ami ± P4 (± IL-1β) treatment during TC with (N=3) or without (N=2) subsequent measurements for oxytocin-stimulated contractions.

**^‡^** All t=0 lengths for tissue strips were measured while they lay straight (without stretch) and flat in saline prior to immersing them in TC media by vertical suspension with a glass bead weight attached.

**^§^** Stress for each tissue strip was calculated using the weight of the glass bead to which they were attached during 24 h TC, which were corrected for buoyancy effect that reduced their actual weight in DMEM to 60 % of their dry weight.

**^¶^** Strain (ΔL/L) was calculated relative to t=0 tissue strip lengths for 24 h TC with simultaneous ITM because no ITM slack length measurements were recorded, in order to minimise disruption within the TC incubator during setup of experiments.
